# CircZSWIM6 mediates dysregulation of ECM and energy homeostasis in ageing chondrocytes through RPS14 post‐translational modification

**DOI:** 10.1002/ctm2.1158

**Published:** 2023-01-05

**Authors:** Zhe Gong, Kefan Wang, Junxin Chen, Jinjin Zhu, Zhenhua Feng, Chenxin Song, Zheyuan Zhang, Haoming Wang, Shunwu Fan, Shuying Shen, Xiangqian Fang

**Affiliations:** ^1^ Department of Orthopaedic Surgery Sir Run Run Shaw Hospital Medical College of Zhejiang University Hangzhou Zhejiang China; ^2^ Key Laboratory of Musculoskeletal System Degeneration and Regeneration Translational Research of Zhejiang Province Hangzhou Zhejiang China; ^3^ Sir Run Run Shaw Institute of Clinical Medicine of Zhejiang University Hangzhou Zhejiang China

**Keywords:** AMPK, CircZSWIM6, metabolism, osteoarthritis, RPS14, senescence

## Abstract

**Background:**

Circular RNAs (CircRNAs) are important and have different roles in disease progression. Herein, we aim to elucidate the roles of a novel CircRNA (CircZSWIM6) which is upregulated in ageing chondrocytes.

**Methods:**

We verified the roles of CircZSWIM6 in senescent and osteoarthritis (OA) development in vitro through CircZSWIM6 knockdown and overexpression. RNA pulldown assay and RNA binding protein immunoprecipitation were performed to identify the interaction between CircZSWIM6 and Ribosomal protein S14 (RPS14). The roles of CircZSWIM6 in ageing‐related OA were also confirmed in non‐traumatic and traumatic model respectively.

**Results:**

CircZSWIM6 regulates extracellular matrix (ECM) and energy metabolism in ageing chondrocyte. Mechanistically, CircZSWIM6 competitively bound to the E3 ligase STUB1 binding site on RPS14 (K125) to inhibit proteasomal degradation of RPS14 to maintain RPS14 function. CircZSWIM6‐RPS14 axis is highly associated with AMPK signaling transduction, which keeps energy metabolism in chondrocyte. Furthermore, CircZSWIM6 AAV infection leads to senescent and OA phenotypes in a non‐traumatic model and accelerates OA progression in a traumatic model.

**Conclusion:**

Our results revealed a significant role of CircZSWIM6 in age‐related OA by regulating ECM metabolism and AMPK‐associated energy metabolism. We highlight the CircZSWIM6‐RPS14‐PCK1‐AMPK axis is a potential biomarker for OA.

## Graphical abstract

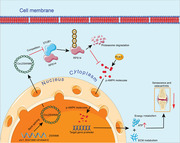

## 1 INTRODUCTION

Homeostasis of cartilage matrix is maintained by cartilage cells. The extracellular matrix (ECM) is a major component in cartilage and is important for chondrocyte function. In addition to supporting chondrocyte growth, the ECM responds to external environmental stimuli through dynamic regulation to maintain articular cartilage homeostasis.^1^ However, disruption or damage to cartilage causes an imbalance in ECM metabolism, leading to osteoarthritis (OA).^2^ OA is highly associated with age, particularly in the elderly.^3^ It usually manifests as joint pain, joint stiffness and movement disorders and eventually leads to a decline in quality of life. Cumulative evidence indicates that OA is associated with ageing, obesity, gender, strain, and trauma, among which ageing is the most important factor.^4^ Hence, investigating the mechanisms underlying ageing and OA has been a primary focus of research.

Cartilage damage, synovial inflammation and subchondral osteosclerosis are the main characterizations of OA.^5^ Age‐related pathogenesis leads to chondrocyte senescence and imbalance in ECM metabolism, resulting in an increase in a variety of ECM‐degrading enzymes such as MMP13 and ADAMTS5. Senescent chondrocytes are highly express specific cyclin‐dependent kinases (CDK) inhibitors, such as CDKN2A and CDKN1A, with low growth activity.^6^ Meanwhile, the senescence of chondrocytes contributes to the expression of nuclear γ‐H2AX foci, a marker for DNA damage responses.^7^ To date, studies have focused primarily on the rejuvenation of senescent cells. Among potential therapeutic approaches, targeted clearance of senescent cells has attracted extensive attention.^8^ The removal of senescent or damaged cells attenuates OA progression in a mouse model.^9^ Elimination of ageing cells was found to improve physiological function and exercise ability in old mice.^10^ However, the potential molecular pathways that regulate senescence phenotypes have not been thoroughly studied. Therefore, elucidating the molecular mechanisms underlying senescence in chondrocytes could represent a breakthrough for further investigation.

In addition to ECM metabolism dysregulation, energy metabolism imbalance is also involved in age‐related OA progression.^11^ With age, articular chondrocytes exhibit a decreased capacity to synthesize adenosine triphosphate (ATP) and maintain their production.^12^ AMP‐activated protein kinase (AMPK) is associated with ATP production,^13^ which participates in energy balance and metabolism.^14^ AMPK dysregulation contributes to age‐related diseases.^15^, ^16^ A reduction in AMPK phosphorylation has been observed in human and mouse OA models.^17^ OA chondrocytes show the decreased phosphorylation of AMPK compared to normal chondrocytes.^18^ Therefore, the maintenance of AMPK activity is vital for energy homeostasis in chondrocytes. Ribosomal protein S14 (RPS14) is associated with cellular senescence, which unites the MDM2‐p53 loop reported in a previous study.^19^ The relationship between RPS14 and age‐related OA is not well understood. One study reported that the upregulation of RPS14 was observed in OA samples,^20^ which suggests that RPS14 may participate in OA development. Moreover, the relationship between RPS14 and energy metabolism in chondrocytes has not been studied. This study aims to explore the molecular mechanisms involved in RPS14 and age‐related OA development, which is crucial for treating OA.

Unlike linear mRNAs cleaved in the classical pathway, circular RNAs (circRNAs) show a closed‐ring molecule which is formed by back‐splicing of the free 3′ or 5′ ends^21^ in circRNAs that does not contain polar or polyadenylated tails. CircRNAs have various functions in various diseases.^22^, ^23^ We previously reported that circRNA‐targeting microRNAs (miRNAs) can alleviate OA degree.^24^ CircRNAs also combine with specific proteins in OA progression or other diseases.^5^, ^25^, ^26^ Although most circRNAs are non‐coding, certain circRNAs with the capability to code for proteins have recently been identified.^27^ These characteristics demonstrate that circRNAs represent ideal biomarkers for many diseases.

A novel circRNA, termed CircZSWIM6, was detected in ageing chondrocytes produced by ZSWIM6 (hsa_circ_0006381). CircZSWIM6 enhanced RPS14 stability by competitively inhibiting STUB1‐mediated ubiquitination. The CircZSWIM6‐RPS14 axis inhibited phoenolpyruvate carboxykinase 1 (PCK1)‐related AMPK activation and decreased ATP production in ageing chondrocytes. We believe that our study indicates that the CircZSWIM6–RPS14–PCK1–AMPK axis is a potential target for OA.

## 2 MATERIALS AND METHODS

### 2.1 Collection of human knee joint samples

Human cartilage samples were collected at Sir Run Run Shaw Hospital (Zhejiang, China) with approval from the Ethics Committee. Knee specimens aged 45–55 years were considered the relatively younger group, whereas specimens aged 75–85 years were considered the relatively old group. In the younger and older groups, medial tibial plateau tissues were used as the damaged group, whereas relatively undamaged lateral tibial plateau tissues were used as controls. Descriptive characteristics of human cartilage samples are listed in Table S2.

### 2.2 Cell culture

Samples of knee cartilage were minced under sterile condition and digested overnight with collagenase at 37°C. After filtering through a 200 mm mesh and centrifuging for 5 min at 800 rpm, the cells were cultured in Dulbecco's modified Eagle's medium (DMEM) containing 4.5 g/L d‐glucose, l‐glutamine, and 110 mg/L sodium pyruvate (Gibco, cat. no. 11965092), supplemented with 1% penicillin/streptomycin (Thermo Fisher, cat. no. 15140122) and 10% foetal bovine serum (Gibco, cat. no. 10099141C). Chondrocytes were incubated in an incubator containing 5% CO_2_ at 37°C.

Following a previous study, we isolated mouse chondrocytes from 5‐day‐old C57BL/6 mice's femoral condyles and tibial plateaus for primary culture.^28^ The mice were sacrificed under general anaesthesia. In sterile conditions, the anterior legs were fixed with needles, and the hind legs’ skin and soft tissues were removed. The translucent parts of the femoral condyles and tibial plateau were then dislocated, and soft tissues were removed thoroughly under a microscope. Next, cartilage pieces were cut into small pieces and placed in a new petri dish with collagenase digestion solution overnight at 37°C. The cell suspension was filtered and centrifuged at room temperature for 5 min at 800 rpm after digestion. Chondrocyte suspensions were cultured in high‐glucose DMEM and maintained under sterile conditions in a 37°C incubator under 5% CO_2_.

### 2.3 RNA sequencing

RNA‐seq analyses were performed from a mixture of primary chondrocytes from five younger (45–55 years) individuals and a mixture of primary chondrocytes from five older individuals (75–85 years) according to our previous study.^29^ RNA sequencing was supported by RiboBio Technology (Guangzhou, China). Significant differences were defined as a |log2FC(older/younger)| > 1 and FDR > .05.

### 2.4 Chondrocyte treatment

For the chondrocyte senescence model, doxorubicin (Doxo) (100 nM, Selleck, cat. no. S1208, China) was used. We treated chondrocyte cells with or without Doxo for 5 days, with Doxo‐supplemented media being refreshed every 2 days. Chondrocytes were treated with bortezomib (Borz) (250 nM, Selleck, cat. no. S1013, China) to inhibit proteasomes. Chondrocytes were treated with cycloheximide (10 μg/ml, Selleck, cat. no. S7418, China) to inhibit protein synthesis. Chondrocytes were treated with dorsomorphin (10 μM; MedChemExpress, cat. no. HY‐13418A, China) to inhibit AMPK activation.

### 2.5 Senescence β‐galactosidase (SA‐β‐Gal) staining and Alcian blue staining

The senescence β‐galactosidase (SA‐β‐Gal) staining kit (Beyotime, cat. no. C0602, China) was used to detect senescent chondrocytes according to the manufacturer's instructions. We observed SA‐β‐Gal cells using an inverted phase contrast microscope (Lecia, Germany). Total cells and SA‐β‐Gal‐positive cells were counted per culture dish.

Cells or tissues were incubated with 1% Alcian blue solution for 1 h. After incubation, cells or tissues were washed twice with PBS. An inverted phase‐contrast microscope (Leica, Germany) was used to obtain representative images.

### 2.6 Western blot

Cell samples were collected and lysed in radioimmunoprecipitation assay lysis buffer containing 50 mM Tris (pH 7.4), 150 mM NaCl, 1% Triton X‐100, 1% sodium deoxycholate, .1% sodium dodecyl sulphate, sodium orthovanadate, sodium fluoride and 1 mM EDTA (Fude Biological Technology, cat. no. FD009, China) and a mixture of phosphatase and protease inhibitor cocktails (Thermo Fisher, cat. no. 78440). Protein solutions were obtained and quantified by using the BCA assay (Beyotime, cat. no. P0010S, China). SDS–PAGE was used to separate the protein samples, which were then transferred to a polyvinylidene fluoride membrane. After blocking with 5% skim milk (cat. no. FD0080, China) for 2 h, primary antibodies were incubated overnight on the membrane, followed by a secondary antibody conjugated to horseradish peroxidase for 1 h at room temperature. The information of primary antibodies are shown in Table S5. Unbound primary antibodies were removed using TBST buffer containing Tris–HCl, NaCl and Tween 20 (Fude Biological Technology, cat. no. FD9058, China). Immunoreactive protein bands were detected using ECL substrate (Fude Biological Technology, cat. no. FD8020, China).

### 2.7 Histology and immunofluorescence

Human knee joint tissues after were embedded in paraffin wax and sectioned to 5 μm. Then, sections were incubated in Fast Green solution (Sigma, cat. no. F7252, USA) for 3 min. Following thorough washing with tap water, the sections were incubated for 10 s with Safranin‐O solution (Sigma, cat. no. S8884, USA). For Alcian blue staining, cells were incubated in an Alcian blue solution (Sigma, cat. no. 33864‐99‐2, USA) for 1 h. Osteoarthritis Research Society International (OARSI) grading system (0–6) was used to evaluate OA severity.

Sections and cells permeabilized by Triton X‐100 solution (Sigma, cat. no. T8787, USA), followed by incubation with 5% BSA for 2 h. After that, the sections and cells were incubated overnight at 4°C with primary antibodies (Table S5). After incubation, the primary antibodies were washed three times in PBS to remove unbound antibodies. It was then incubated with Alexa 488–conjugated goat anti‐mouse secondary antibodies (1:500, Beyotime, cat. no. A0428, China) and Alexa 555‐conjugated donkey anti‐rabbit secondary antibodies (1:500, Beyotime, China). DAPI (Invitrogen) was used as a stain for nuclei. An inverted fluorescence microscope (Leica) was used to observe cell samples and scan sections using a fluorescence microscope (Leica).

### 2.8 RNA extraction and RT‐qPCR experiment

Human or mouse chondrocytes were treated with RNAiso reagent (TaKaRa Bio, Japan) to extract total cellular RNA. PrimeScript RT Reagent Kit (Accurate Biotechnology, Hunan, China) was used for reverse transcription. Reaction volumes containing amplification primers and UltraSYBR Mixture (Yeason, China) were determined using an ABI 7500 Sequencing Detection System (Applied Biosystems, Foster City, CA, USA) to detect amplification products of specific circRNAs or mRNAs. β‐Actin (*ACTB*) rRNA was chosen as a housekeeping gene. In Table S1, we list all PCR primer sequences used in this study.

### 2.9 Co‐immunoprecipitation (Co‐IP)

As soon as the plasmid transfection was complete, the cells were lysed in IP lysis buffer (20 mM Tris–HCl [pH 7.4], 150 mM NaCl, 1 mM EDTA, 1% Nonidet P40 and 1 mM PMSF). The protein was obtained after centrifugation followed by IP with anti‐tag antibodies and Sepharose beads (Accurate Biotechnology) at 4°C overnight. Anti‐tag antibodies are listed in Table S5. Immediately following incubation, immune complexes were washed with NaCl buffer three times and incubated in SDS loading buffer for 1 h at 95°C. Proteins of the IPs were analysed by western blotting.

### 2.10 Small interfering RNA transfection

The knock‐down of CircZSWIM6 or specific mRNAs was achieved using small interfering RNA (siRNA). SiRNAs (RiboBio, Guangzhou, China) were designed and transfected into chondrocytes to detect the knock‐down efficiency of CircZSWIM6 or specific mRNAs. Thermo Fisher cat. no. 13778075 Lipofectamine RNAiMAX transfection reagent was used for siRNA transfection. Table S1 lists all siRNA sequences.

### 2.11 RNA pull‐down assay

CircZSWIM6‐binding proteins were detected using an RNA pull‐down kit (BersinBio, China). Biotin‐labelled CircZSWIM6 and LacZ probes (control probe) were designed by Shanghai GenePharma Co., Ltd. Briefly, 10^7^ human chondrocytes were collected and lysed with IP lysis buffer. The supernatant of 100 μl was collected as input, In the remaining supernatant, either CircZSWIM6‐specific probe streptavidin Dynabeads mixture or a control probe streptavidin Dynabeads mixture was incubated overnight at 4°C. After incubation, non‐specifically bound material was removed by washing with lysis buffer. Specifically bound proteins were obtained using protein eluent buffer containing glycine‐HCl and dithiothreitol (BersinBio, China), and the supernatant was used for mass spectrometry (MS) identification. Table S1 lists the sequence of the CircZSWIM6 probe.

### 2.12 RNA‐binding protein immunoprecipitation (RIP)

Assays were performed using the RIP Kit (BersinBio, China) according to the manufacturer's instructions. The co‐precipitated RNAs were then detected by quantitative real‐time polymerase chain reaction (RT‐qPCR).

### 2.13 Plasmid

For CircZSWIM6 overexpression, the human CircZSWIM6 linear sequence was synthesized and subcloned into pHBAd‐circRNA (HanBio, Shanghai, China). For RPS14 overexpression, human *RPS14* cDNA was synthesized and subcloned into the pcDNA vector (v.3.0) with a FLAG tag. For the RNA immunoprecipitation (RIP) assay, linear CircZSWIM6 and its fragments were synthesized and subcloned into the pcDNA v.3.0 vector with a FLAG tag. Meanwhile, RPS14 fragments, according to their protein domain, were synthesized and subcloned into the pcDNA v.3.0 vector with a FLAG tag. For the IP assay, full‐length RPS14, STUB1 and UB plasmids were acquired by cloning cDNAs into pcDNA v.3.0, with a corresponding haemagglutinin or FLAG tag.

### 2.14 Nile red staining

Chondrocytes transfected with CircZSWIM6 siRNA, RPS14 siRNA or the RPS14 plasmid were fixed with 4% PFA. Chondrocytes were incubated with 1 μM Nile red solution (MedChemExpress, cat. no. HY‐D0718, China) for 20 min.

### 2.15 ATP detection

Chondrocytes were collected after treatment. The cells were then lysed on ice. After centrifugation, the supernatant was added to the substrate solution, followed by detection using an illuminometer at intervals of 10 s per well according to the manufacturer's instructions (Beyotime, cat. no. S0026, China).

### 2.16 Oxygen consumption rate (OCR) analysis

In order to measure the oxygen consumption rate (OCR), Seahorse XF Cell Mito Stress Test kits and Bioscience XF96 Extracellular Flux Analyzers were used. Briefly, chondrocytes were seeded in Seahorse XF96 plates with glucose‐containing media at 1 × 10^4^ cells/well and incubated overnight. The OCR was measured using chondrocytes that had been treated with oligomycin, FCCP and rotenone. OCR data were analysed using Wave software and plotted as means ± SD.

### 2.17 Animal model

An Institutional Animal Care and Use Committee of the Institute of Health Sciences (Zhejiang, China) approved all animal experiments. A diagram of animals used and treatment is listed in Table S4. We divided mice into three groups for the non‐traumatic model: sham, sham + vector and sham + CircZSWIM6. For a traumatic model, the mice were divided into three groups: sham, DMM, and DMM + CircZSWIM6 (*n* = 6). For the DMM operation, animals were anaesthetized with 1% sodium pentobarbital. The medial parapatellar approach was used for the DMM operation. Under a microscope, a medial meniscus was removed after exposing the knee joint. A 5‐day course of buprenorphine (.05 mg/kg) was given to all animals after the wounds were closed. Animals in the sham group underwent capsulotomy without medial meniscal resection. Mice in the sham + vector and DMM + CircZSWIM6 groups received a total of 10 μl of CircZSWIM6 or adeno‐associated virus vector (AAV, approximately 1 × 10^12^ vg/ml) created and packed by HanBio (Shanghai, China). Two months after AAV infection, all animals were sacrificed, and knee articular tissue was harvested for pathological and micro‐CT analyses.

### 2.18 Statistical analysis

Prism GraphPad 8.0 software was used for all statistical analyses. ANOVA or Student's *t*‐test are used to analyse data, followed by Tukey's or Dunnett's T3 post hoc tests for multiple comparisons. *p* < .05 was used to determine statistical significance.

## 3 RESULTS

### 3.1 Validation of senescent phenotypes in the older human cohort

**FIGURE 1:**
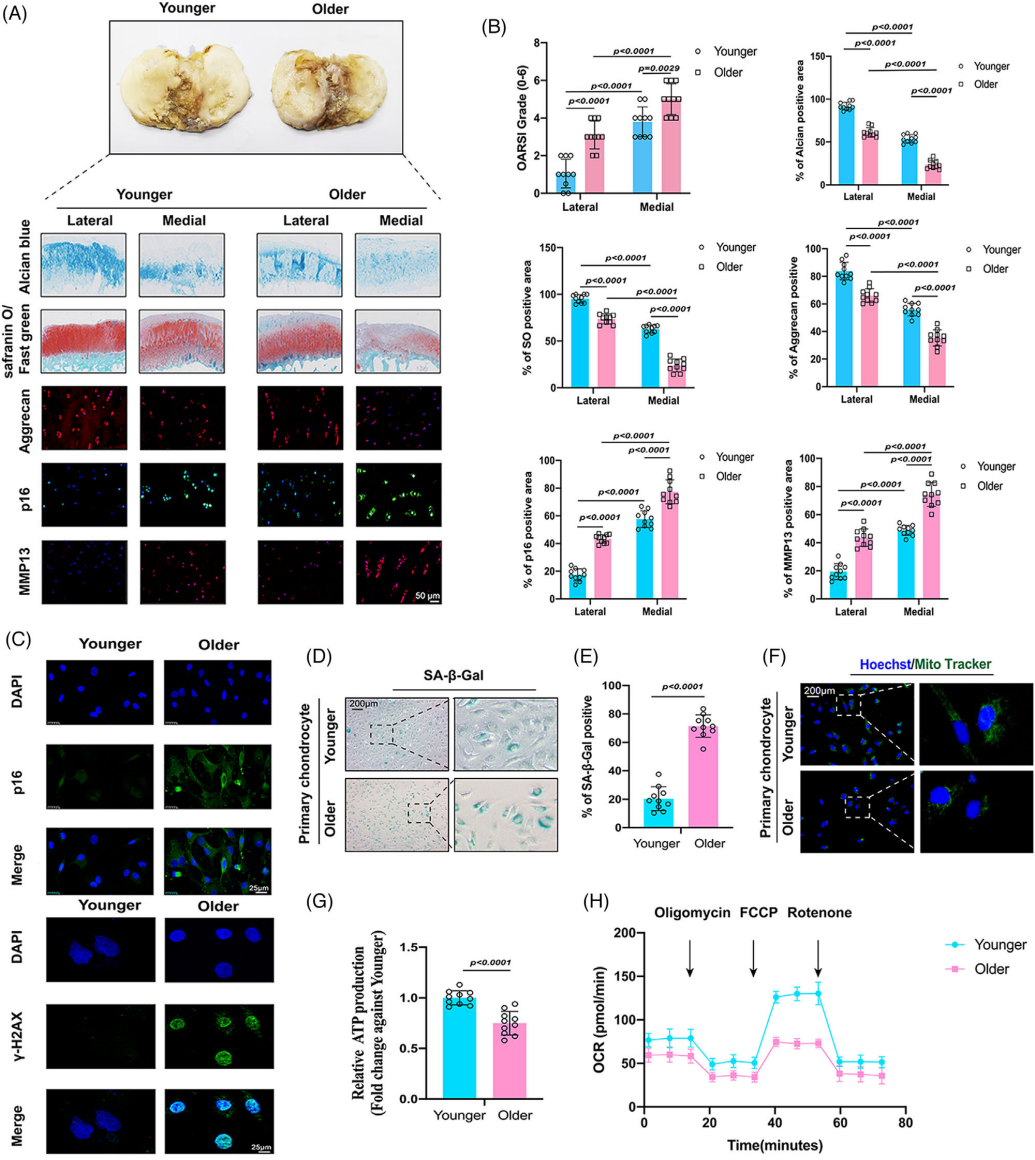
Validation of senescent phenotypes in the older human cohort: (A) representative images of Safranin‐O/Fast green staining, Alcian blue staining and immunofluorescence of aggrecan, p16 and MMP13 between younger group (45–55‐year old) and older group (75–85‐year old); (B) quantifications of Osteoarthritis Research Society International (OARSI) grades, aggrecan positive staining, SO‐positive staining, Alcian blue–positive staining, MMP13 positive staining and p16 positive staining; (C) representative immunofluorescence images of p16 and γ‐H2AX in younger and older chondrocytes; (D) senescence β‐galactosidase (SA‐β‐Gal) staining in younger and older chondrocytes; (E) quantification of SA‐β‐Gal staining in younger and older chondrocytes; (F) MitoTracker staining in younger and older chondrocytes; (G) quantification of adenosine triphosphate (ATP) production in younger and older chondrocytes; (H) oxygen consumption rate (OCR), which reflects mitochondrial respiration, was decreased in older chondrocytes; quantitative data shown as mean ± SD; exact *p*‐values are shown in figures. Two‐side unpaired Student's *t*‐test was used for statistical analysis. One‐way ANOVA with Tukey's multiple comparison was used for statistical analysis.

We collected data on the knee articular joints of patients who underwent total knee joint replacement. Patients aged 45–55 years were considered younger, whereas those aged 75–85 years were considered older. Medial cartilage of older individuals had significantly decreased cartilage matrix and thickness compared with those in lateral and younger cartilages (Figure 1A). Representative immunofluorescence images indicated that the expression of matrix synthases, such as aggrecan, was lower in older patients than in younger patients, whereas the expression of matrix decomposing enzymes, such as MMP13, and CDK inhibitors, such as p16, was significantly higher in older patients than in younger patients (Figure 1A). Quantification of these pathological analyses, together with the OARSI scoring system, reflected similar findings (Figure 1B). Representative immunofluorescent images also showed an increased expression of p16 and the DNA damage marker γ‐H2AX in older chondrocytes (Figure 1C). In addition, more SA‐β‐Gal‐positive areas were observed in the older chondrocytes (Figure 1D). Quantification of SA‐β‐Gal positive areas confirmed this result (Figure 1E). A reduction in functional mitochondria was observed in older chondrocytes using MitoTracker staining (Figure 1F), accompanied by a reduction in ATP production (Figure 1G). In addition, OCR analysis indicated a decreased OCR in older chondrocytes compared to younger chondrocytes (Figure 1H). Altogether, these findings suggested obvious senescence and OA phenotypes in older knee articular cartilage and chondrocytes.

### 3.2 CircZSWIM6 is highly expressed in ageing chondrocytes

**FIGURE 2:**
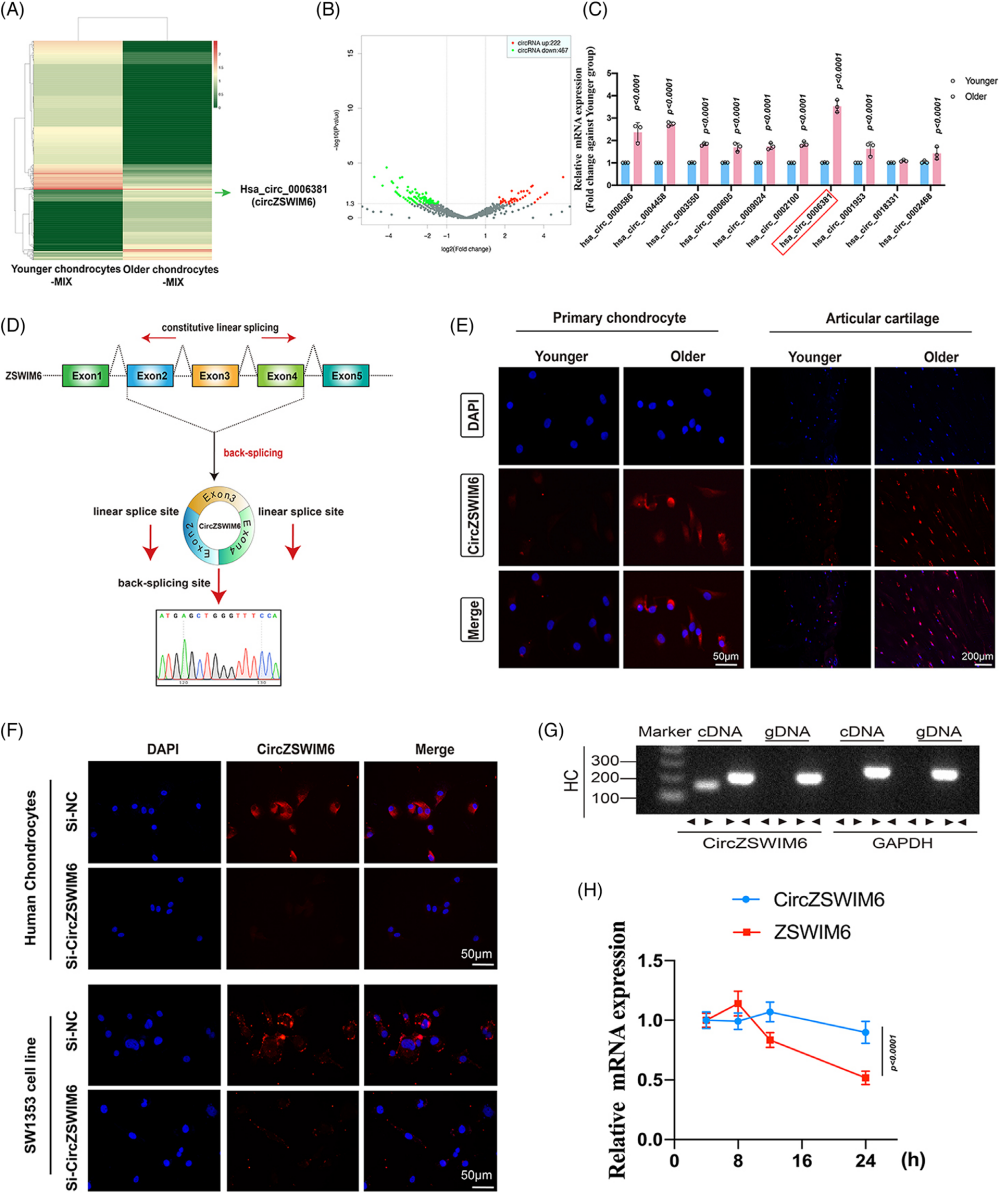
Identification and characterization of CircZSWIM6: (A) A heat map between younger chondrocytes‐MIX and older chondrocytes‐MIX. Has_circ_0006381 (CircZSWIM6) is upregulated in older chondrocyte; (B) a volcano map after RNA deep sequence; (C) CircZSWIM6 is highly expressed in older chondrocytes indicated by quantitative real‐time polymerase chain reaction (RT‐qPCR) (*n* = 3); (D) schematic illustration showing ZSWIM6 exons 2–4 circularization to form CircZSWIM6. The presence of CircZSWIM6 is confirmed by Sanger sequencing; (E) CircZSWIM6 expression among younger (45–55‐year old) and older (75–85‐year old) primary chondrocytes or knee articular samples revealed by fluorescent in situ hybridization (FISH) staining. CircZSWIM6 probe is labelled with Cy3; (F) FISH staining reveals CircZSWIM6 predominantly locates in cytoplasm while CircZSWIM6 knock‐down significantly decrease its expression in cytoplasm in HCs and SW1353 cells; (G) divergent primers amplified CircZSWIM6 from cDNA instead of genomic DNA; (H) HCs treated with actinomycin D for 8, 16 and 24 h and half‐life period of CircZSWIM6 is longer than host gene *ZSWIM6* indicated by RT‐qPCR (*n* = 3, three donors for three replicates). Quantitative data shown as mean ± SD; exact *p*‐values are shown in figures. Two‐side unpaired Student's *t*‐test was used for statistical analysis. One‐way ANOVA with Tukey's multiple comparison was used for statistical analysis.

RNA‐seq analysis was performed using RiboBio Technology (Guangzhou, China). In total, 7306 circRNAs were identified by RNAseq analysis (Figure 2A). A total of 689 circRNAs that were differentially expressed in older and younger chondrocytes were identified by RNA‐seq mapping to the reference genome (hg38, human genome), with |log2FC(older/younger)| > 1 and FDR ≤ .05, among which 222 circRNAs were significantly upregulated and 467 circRNAs were significantly downregulated (Figure 2B). We selected the top‐10 upregulated circRNAs for further investigation (hsa_circ_0000586, hsa_circ_0004458, hsa_circ_0003550, hsa_circ_0000605, hsa_circ_0009024, hsa_circ_0002100, hsa_circ_0006381, hsa_circ_0001953, hsa_circ_0018331 and hsa_circ_0002468). Next, we performed RT‐qPCR quantification to verify the top 10 circRNAs, showing that CircZSWIM6 (hsa_circ_0006381) levels were significantly increased in older chondrocytes (Figure 2C).

### 3.3 CircZSWIM6 expression and characterizations in ageing progression

A fluorescent in situ hybridization (FISH) analysis of CircZSWIM6 expression levels in primary chondrocytes and articular tissues of different ages was used to investigate CircZSWIM6's role in ageing. CircZSWIM6 expression was higher in older chondrocytes (75–85 years) and articular cartilage (Figure 2E). These data revealed that CircZSWIM6 is involved in chondrocyte senescence progression.

The CircZSWIM6 junction was confirmed using Sanger DNA sequencing (Figure 2D). The CircZSWIM6 gene was amplified only using divergent primers on cDNA, and there was no amplification product observed on gDNA (Figure 2G). Moreover, FISH staining indicated that CircZSWIM6 was abundant in the cytoplasm of chondrocytes and SW1353 cells (Figure 2F). However, when CircZSWIM6 was knocked down, its cytoplasmic expression was significantly decreased (Figure 2F). Furthermore, actinomycin D, an inhibitor of transcription, was co‐cultured with chondrocytes for 8, 16 and 24 h. RT‐qPCR results indicated that the half‐life period of CircZSWIM6 was longer than that of the host gene *ZSWIM6* (Figure 2H).

### 3.4 CircZSWIM6 regulates senescent phenotypes and extracellular matrix metabolism in chondrocytes

**FIGURE 3:**
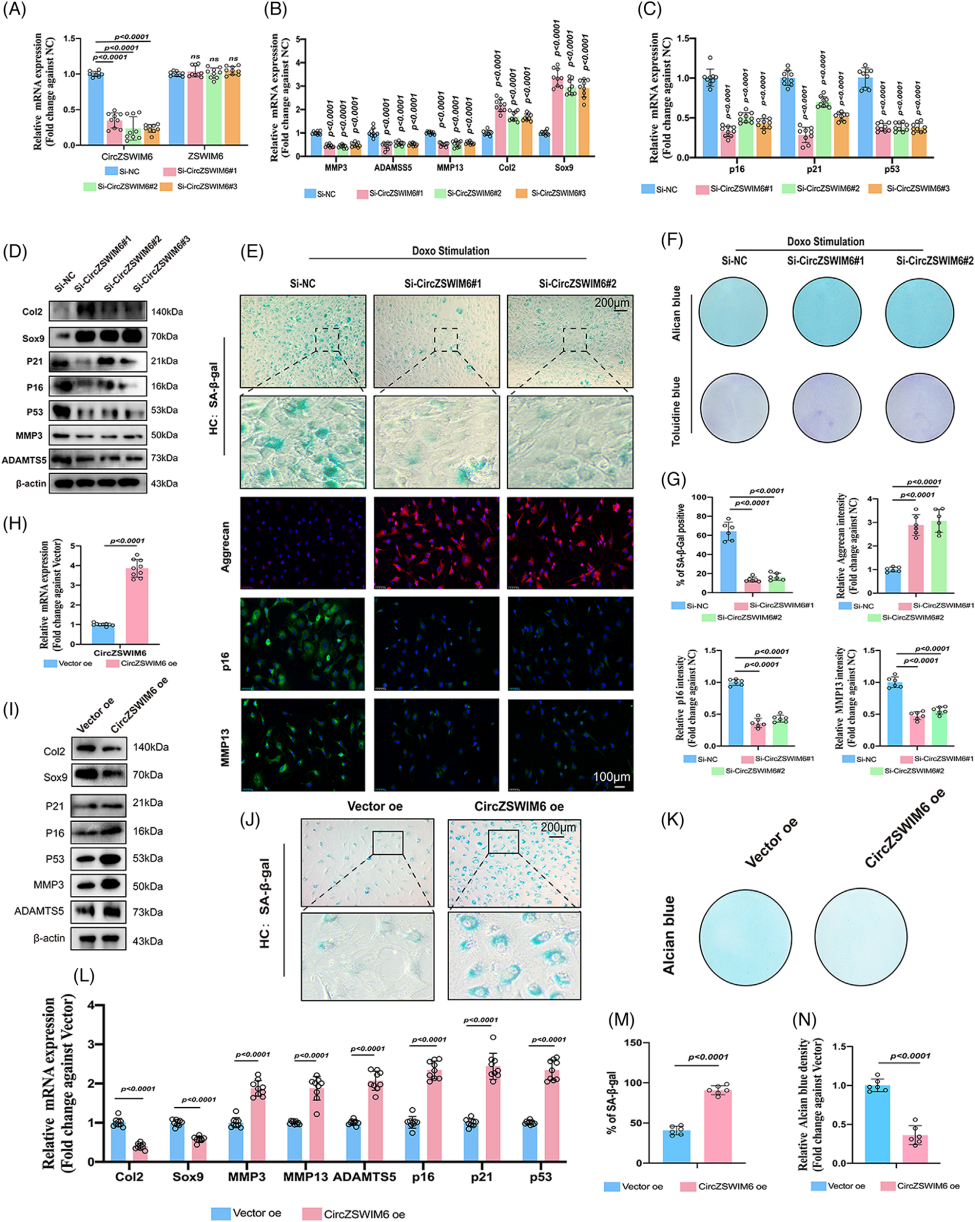
CircZSWIM6 knock‐down or overexpression governs extracellular matrix metabolism and senescent phenotype: (A) human chondrocyte transfected with three CircZSWIM6 small interfering RNA (siRNA); knock‐down efficiency indicated by quantitative real‐time polymerase chain reaction (RT‐qPCR) (*n* = 3, three donors for three replicates); no change of ZSWIM6 mRNA expression is detected; (B and C) mRNA level of *Col2*, *Sox9*, *MMP13*, *ADAMTS5*, *p16*, *p21* and *p53* in human chondrocyte with CircZSWIM6 knock‐down (*n* = 3, three donors for three replicates); (D) protein level of Col2, Sox9, p21, p16, p53, MMP3 and ADAMTS5 in human chondrocyte after CircZSWIM6 knock‐down; (E) senescence β‐galactosidase (SA‐β‐Gal) staining, immunofluorescence images of aggrecan, p16 and MMP13 in chondrocytes after CircZSWIM6 knock‐down. (F) Alcian blue and Toluidine blue staining in human chondrocyte with CircZSWIM6 knock‐down, (G) SA‐β‐Gal of human chondrocyte with CircZSWIM6 knock‐down or doxorubicin stimulated human chondrocyte with or without CircZSWIM6 knock‐down: quantification of SA‐β‐Gal, aggrecan, p16 and MMP13 stainings (*n* = 6); (H) CircZSWIM6 overexpression efficiency (*n* = 3, three donors for three replicates); (I) protein level of Col2, Sox9, p21, p16, p53, MMP13 and ADAMTS5 in human chondrocyte with CircZSWIM6 overexpression; (J) SA‐β‐Gal staining in chondrocyte after CircZSWIM6 overexpression; (K) Alcian blue staining in chondrocyte after CircZSWIM6 overexpression; (L) mRNA level of *Col2*, *Sox9*, *p21*, *p16*, *p53*, *MMP13* and *ADAMTS5* in human chondrocyte with CircZSWIM6 overexpression; (M and N) quantifications of SA‐β‐Gal and Alcian blue staining after CircZSWIM6 overexpression; quantitative data shown as mean ± SD; exact *p*‐values are shown in figures. Two‐side unpaired Student's *t*‐test was used for statistical analysis. One‐way ANOVA with Tukey's multiple comparison was used for statistical analysis.

We transfected CircZSWIM6 siRNAs into HCs and MCs, respectively. This significantly decreased the expression of CircZSWIM6/CircZSWIM6 in HCs and MCs, respectively, whereas the knock‐down of CircZSWIM6/CircZSWIM6 did not affect *ZSWIM6/Zswim6* mRNA levels (Figures 3A and S1b).

Next, we examined the effects of CircZSWIM6/CircZSWIM6 on senescence phenotypes and ECM metabolism in HC and MCs. Their knock‐down in HC/MCs significantly decreased the expression of ECM degrading‐related MMP13 and ADAMTS5, as well as senescence associated with p16, p21 and p53, and enhanced Col2 and Sox9 expression, as determined by RT‐qPCR and western blot (Figures 3B–D and S1a,c,d).

Doxo was used to induce chondrocyte senescence according to a previous study.^7^ Chondrocytes treated with different concentrations of Doxo showed less Alcian blue staining and more SA‐β‐Gal positive staining (Figure S2a,b). In addition, chondrocytes cultured with Doxo (100 nM) exhibited the downregulation of Col2 and Sox9 and the upregulation of MMP13, MMP3, ADAMTS5, p16 and p53 protein expression, as indicated by western blot analysis (Figure S2c). Upregulation of CircZSWIM6 expression was confirmed after Doxo stimulation, as indicated by RT‐qPCR (Figure S2d). Knock‐down of CircZSWIM6/CircZSWIM6 decreased SA‐β‐Gal positive staining (Figure S1e), which was more prominent in Doxo‐induced senescent chondrocytes (Figures 3E and S1h). Meanwhile, an inhibition of CircZSWIM6/CircZSWIM6 resulted in increased Alcian blue and toluidine blue staining (Figures 3F and S1f). Representative immunofluorescence images showed that an inhibition of CircZSWIM6 augmented ECM component expressions, such as aggrecan, and reduced matrix‐destructive enzymes, such as MMP13 and senescence marker p16 (Figure 3E). Quantification of these results further confirmed these observations (Figure 3G and S1g,i). These results indicated the important role of CircZSWIM6/CircZSWIM6 in senescence progression.

Next, gain‐of‐function experiments were performed. The CircZSWIM6 plasmid was transfected into HCs, showing CircZSWIM6 upregulation (Figure 3H). CircZSWIM6 overexpression enhanced Col2 and Sox9 downregulation and upregulation of p21, p16, p53, MMP3 and ADAMTS5, as indicated by the western blot analysis (Figure 3I). In addition, CircZSWIM6 overexpression promoted the expression of genes associated with senescence and those of matrix‐destructive enzymes and decreased the gene expression of synthase enzymes, as indicated by RT‐qPCR (Figure 3L). Moreover, CircZSWIM6 overexpression significantly decreased Alcian blue–positive staining (Figure 3K). SA‐β‐Gal staining demonstrated that CircZSWIM6 overexpression in chondrocytes promoted the progression of senescence (Figure 3J). Quantification of SA‐β‐Gal positive staining and Alcian blue staining in CircZSWIM6 overexpression chondrocytes showed the same results (Figure 3M,N). In summary, these data demonstrated that CircZSWIM6 promotes senescent phenotypes and regulates ECM metabolism.

### 3.5 Ribosomal protein S14 (RPS14) interacts with CircZSWIM6 in senescence progression

**FIGURE 4:**
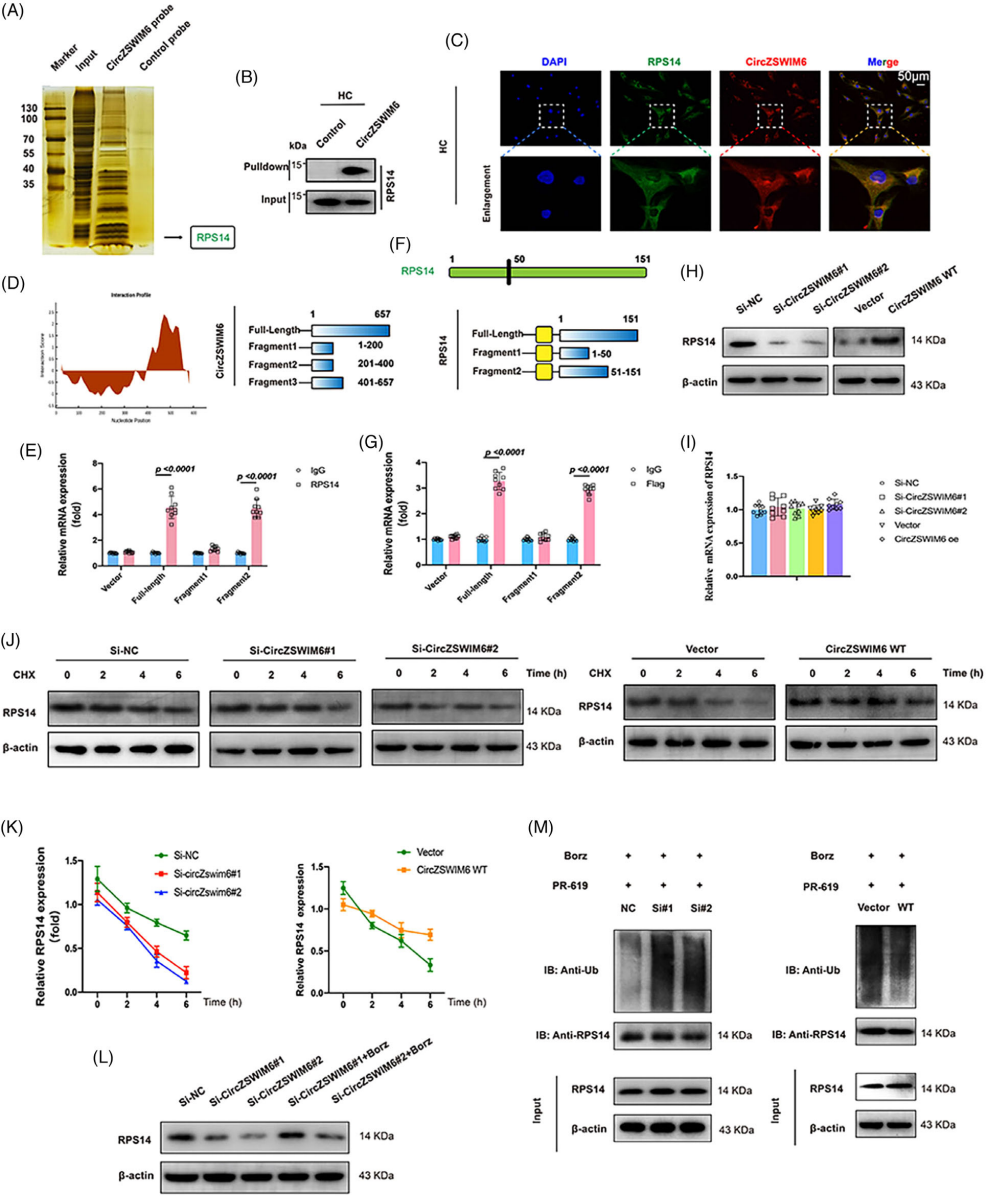
CircZSWIM6 interacts with ribosomal protein S14 (RPS14) and affects RPS14 ubiquitylation: (A) sliver staining of proteins binding to CircZSWIM6; (B) RNA pull‐down is performed using control probe and CircZSWIM6 probe, followed by western blotting using RPS14 antibody; (C) CircZSWIM6 is co‐localized with RPS14 in human chondrocyte; (D) prediction of the regions of CircZSWIM6 interacts with RPS14; (E) binding sequence of RPS14 for CircZSWIM6 identified by RNA immunoprecipitation (RIP) assay; (F) illustration of RPS14 fragments; (G) binding sequence of CircZSWIM6 for RPS14 via RIP assay; (H) RPS14 protein level after CircZSWIM6 knock‐down or overexpression indicated by western blot analysis; (I) mRNA level of RPS14 after CircZSWIM6 knock‐down or overexpression; (J and K) RPS14 protein level in human chondrocyte treated with the transcription inhibitor CHX with or without CircZSWIM6 knock‐down; (L) effect of bortezomib (Borz) on RPS14 protein level after CircZSWIM6 knock‐down; (M) ubiquitylation of RPS14 in human chondrocyte with CircZSWIM6 knock‐down or overexpression; quantitative data shown as mean ± SD; exact *p*‐values are shown in figures. Two‐side unpaired Student's *t*‐test was used for statistical analysis. One‐way ANOVA with Tukey's multiple comparison was used for statistical analysis.

To verify how CircZSWIM6 promotes senescence progression in HCs, an RNA pull‐down assay was performed. Biotin‐labelled CircZSWIM6 probes were used to pull down specific proteins from HCs. The resultant protein solution was tested using MS, which showed that RPS14 interacted with CircZSWIM6 (Figure 4A). Top 20 CircZSWIM6‐binding proteins identified by MS (ranked by prot_score) are shown in Table S3. The interaction between CircZSWIM6 and RPS14 in HC lysates was verified by western blotting after RNA pull‐down (Figure 4B). Consistently, CircZSWIM6 co‐localized with RPS14 in HCs, as indicated by FISH staining (Figure 4C). The binding sites of CircZSWIM6 and RPS14 were predicted using CatRAPID (http://service.tartaglialab.com/page/catrapid_group). Based on the predicted results, we truncated the full‐length CircZSWIM6 into three fragments (F1: 1–200, F2: 201–400 and F3: 401–657) (Figure 4D). Consistent with this prediction, full‐length CircZSWIM6 and F3 were enriched by RPS14 in the RIP assay (Figure 4E). Furthermore, to determine the domain in RPS14 that interacted with CircZSWIM6, we truncated the full length of RPS14 into two fragments with FLAG tags (F1: 1–50 and F2: 51–151) according to methods described in a previous study^19^ (Figure 4F). RIP assays indicated that full‐length RPS14 and F2 interacted with CircZSWIM6 (Figure 4G).

We then examined the role of RPS14 in senescence progression. Two effective RPS14 siRNAs were transfected into HCs, which significantly decreased *RPS14* mRNA expression (Figure S3a). *RPS14* knock‐down increased *Col2*, aggrecan (*ACAN*) and *Sox9* expression and reduced *MMP13*, *ADAMTS5* expression and senescence‐associated genes, as indicated by RT‐qPCR analysis (Figure S3b). Furthermore, RPS14 inhibition showed similar results by western blot analysis (Figure S3c). We then examined senescence phenotypes after *RPS14* knock‐down. *RPS14* knock‐down decreased SA‐β‐Gal‐positive cells and increased Alcian blue–positive cells (Figure S3d,e). Quantification of SA‐β‐Gal and Alcian blue staining demonstrated that *RPS14* knock‐down downregulated the senescence phenotype (Figure S3f,g). Subsequently, gains of function experiments were performed. Transfection with the RPS14 plasmid upregulated *RPS14* mRNA expression in HCs (Figure S3i). RPS14 overexpression increased the expression of matrix‐destructive enzymes and CDK inhibitors, while decreasing the expression of synthesis enzymes, as indicated by RT‐qPCR and western blot analysis (Figure S3h,l). We also found that RPS14 overexpression accelerated senescence through SA‐β‐Gal staining (Figure S3j). These data supported the hypothesis that RPS14 upregulates senescence phenotype procatabolic effects in HCs.

### 3.6 CircZSWIM6 stabilizes RPS14 through inhibition of proteasome‐mediated degradation

Our investigation indicated that the knock‐down or overexpression of CircZSWIM6 regulated RPS14 at the protein level, but not at the mRNA level (Figure 4H,I). This finding suggested that CircZSWIM6 post‐transcriptionally regulates RPS14 expression in chondrocytes. Obvious differences in RPS14 half‐life were observed between the Si‐negative control (NC) and Si‐CircZSWIM6 HCs after cycloheximide (CHX) treatment, whereas CircZSWIM6 overexpression exhibited the opposite effect, suggesting that CircZSWIM6 enhanced RPS14 stability (Figure 4J,K). The ubiquitin–proteasome pathway is involved in protein degradation. To confirm whether these pathways participate in RPS14 degradation, chondrocytes were treated with the proteasome pathway inhibitor Borz. Western blot analysis revealed that the reduction of RPS14 induced by CircZSWIM6 knock‐down was partially reversed by Borz treatment, suggesting that CircZSWIM6 regulated RPS14 levels through proteasomal degradation (Figure 4L). Next, we investigated the polyubiquitination of RPS14. Western blot analysis showed that the polyubiquitination of RPS14 was upregulated after CircZSWIM6 knock‐down and downregulated following CircZSWIM6 overexpression in the endogenous pathway (Figure 4M). In summary, CircZSWIM6 post‐transcriptionally regulated RPS14 stability through the inhibition of the proteasome degradation pathway.

### 3.7 CircZSWIM6 competitively takes over STUB1 binding site on RPS14 to prevent RPS14 proteasomal degradation

**FIGURE 5:**
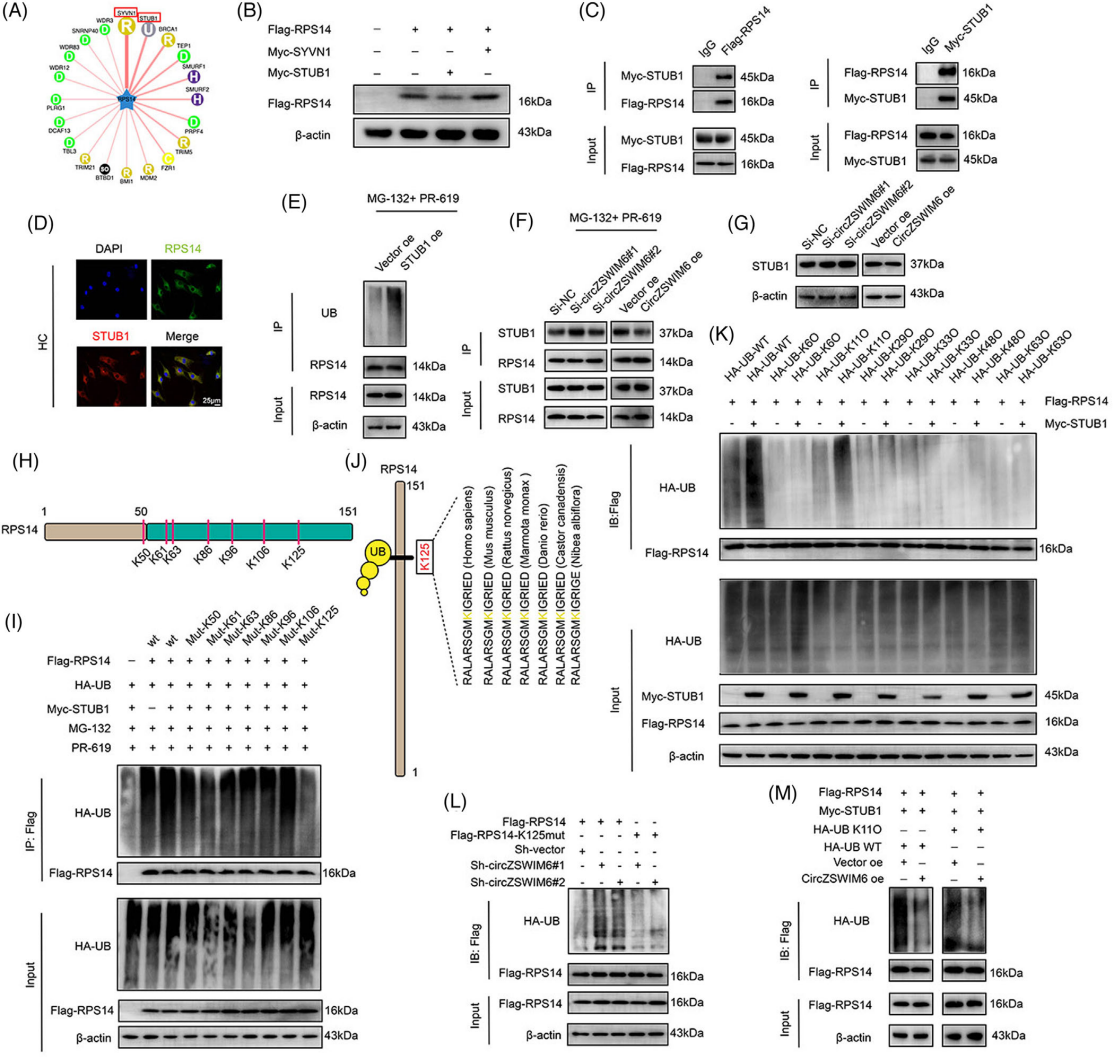
STUB1 is an E3 ligase of ribosomal protein S14 (RPS14), and K125 is the primary ubiquitylation site of RPS14: (A) prediction of potential E3 ligase of RPS14; (B) western blot analysis of FLAG–RPS14 protein level after Myc‐STUB1 and Myc‐SYVN1 transfection, respectively; (C) HEK‐293T cells are transfected with FLAG–RPS14 or Myc‐STUB1. Consecutive immunoprecipitation (Co‐IP) shows interaction between FLAG–RPS14 and Myc‐STUB1. IgG as the negative control; (D) co‐localization of RPS14 (green) and STUB1 (red) in human chondrocyte indicated by immunofluorescence; (E) the ubiquitination level of RPS14 after STUB1 overexpression; (F) effect of CircZSWIM6 knock‐down or overexpression on the interaction between STUB1 and RPS14 in human chondrocyte; (G) western blot analysis of STUB1 protein level after CircZSWIM6 knock‐down or overexpression in human chondrocyte; (H) illustration of ubiquitylation site on RPS14; (I) human chondrocyte transfected with FLAG‐tagged wild‐type or mutant RPS14 KR plasmid and subsequently performed with immunoprecipitation (IP). RPS14 ubiquitylation is tested by western blot analysis; (J) conservation ability of the K125 site of RPS14 among different species; (K) human chondrocyte expressing FLAG–RPS14 and different ubiquitylation types with or without Myc‐STUB1. Ubiquitylation types of RPS14 induced by STUB1 are verified by western blot analysis; (L) effect of CircZSWIM6 knock‐down on the K125R RPS14 ubiquitylation in human chondrocyte; (M) effect of CircZSWIM6 overexpression on K11 ubiquitylation level of RPS14 in human chondrocyte; quantitative data shown as mean ± SD; exact *p*‐values are shown in figures. Two‐side unpaired Student's *t*‐test was used for statistical analysis. One‐way ANOVA with Tukey's multiple comparison was used for statistical analysis.

Using UbiBrowser (http://ubibrowser.ncpsb.org.cn), we predicted that E3 ligases potentially participate in the proteasomal degradation of RPS14 (Figure 5A). We selected two E3 ligases, SYVN1 and STUB1, to investigate their effects on RPS14 protein levels. Myc‐SYVN1 and Myc‐STUB1 plasmids were co‐transfected with Flag‐RPS14 into HCs. Western blot analysis indicated that STUB1 affected RPS14 levels, whereas no change was observed after Myc‐SYVN1 transfection (Figure 5B). Therefore, Myc‐STUB1 and Flag‐RPS14 were co‐transfected into HCs for Co‐immunoprecipitation (Co‐IP) assays. Co‐IP assay results indicated an interaction between RPS14 and STUB1 (Figure 5C). Consistently, RPS14 co‐localized with STUB1 in HCs (Figure 5D). Moreover, STUB1 overexpression promoted RPS14 ubiquitination (Figure 5E). In addition, Co‐IP assays showed that the interaction between STUB1 and RPS14 was enhanced following CircZSWIM6 knock‐down and decreased after CircZSWIM6 overexpression (Figure 5F). However, CircZSWIM6 knock‐down or overexpression did not affect STUB1 levels in chondrocytes (Figure 5G).

Therefore, we investigated potential RPS14 ubiquitylation sites. Through bioinformatic prediction, we identified seven potential ubiquitylation sites on RPS14 (K50, K61, K63, K86, K96, K106 and K125) (Figure 5H). We mutated a conserved RPS14 site to determine the ubiquitylation site on RPS14. IP assays indicated that the K125 mutant of RPS14 significantly decreased ubiquitylation compared to that of the wild‐type (WT), suggesting that K125 is the major ubiquitylation site on RPS14 (Figure 5I). In addition, K125 on RPS14 was highly conserved among different species (Figure 5J).

To confirm the type of ubiquitination involved in the STUB1‐induced proteasomal degradation of RPS14, we transfected different types of UB plasmids (WT, K6, K11, K29, K33, K48 and K63) into HCs. IP assays indicated that the K11 ubiquitin chain participated in RPS14 ubiquitination, and STUB1 overexpression enhanced K11‐induced ubiquitination, indicating that STUB1 contributes to RPS14 ubiquitination through K11‐specific ubiquitination (Figure 5K). In addition, CircZSWIM6 overexpression decreased K11 ubiquitination, similar to that of the WT, indicating that CircZSWIM6 occupied the STUB1 binding site on RPS14 to prevent K11 ubiquitination‐mediated degradation (Figure 5M).

Rescue experiments were performed in the next step. IP assays showed that CircZSWIM6 knock‐down increased RPS14 ubiquitination when the K125 site on RPS14 was not mutated, indicating that CircZSWIM6 took over the K125 site on RPS14 to prevent its proteasomal degradation (Figure 5L).

### 3.8 CircZSWIM6–RPS14 axis regulates AMPK signalling pathway in chondrocytes

**FIGURE 6:**
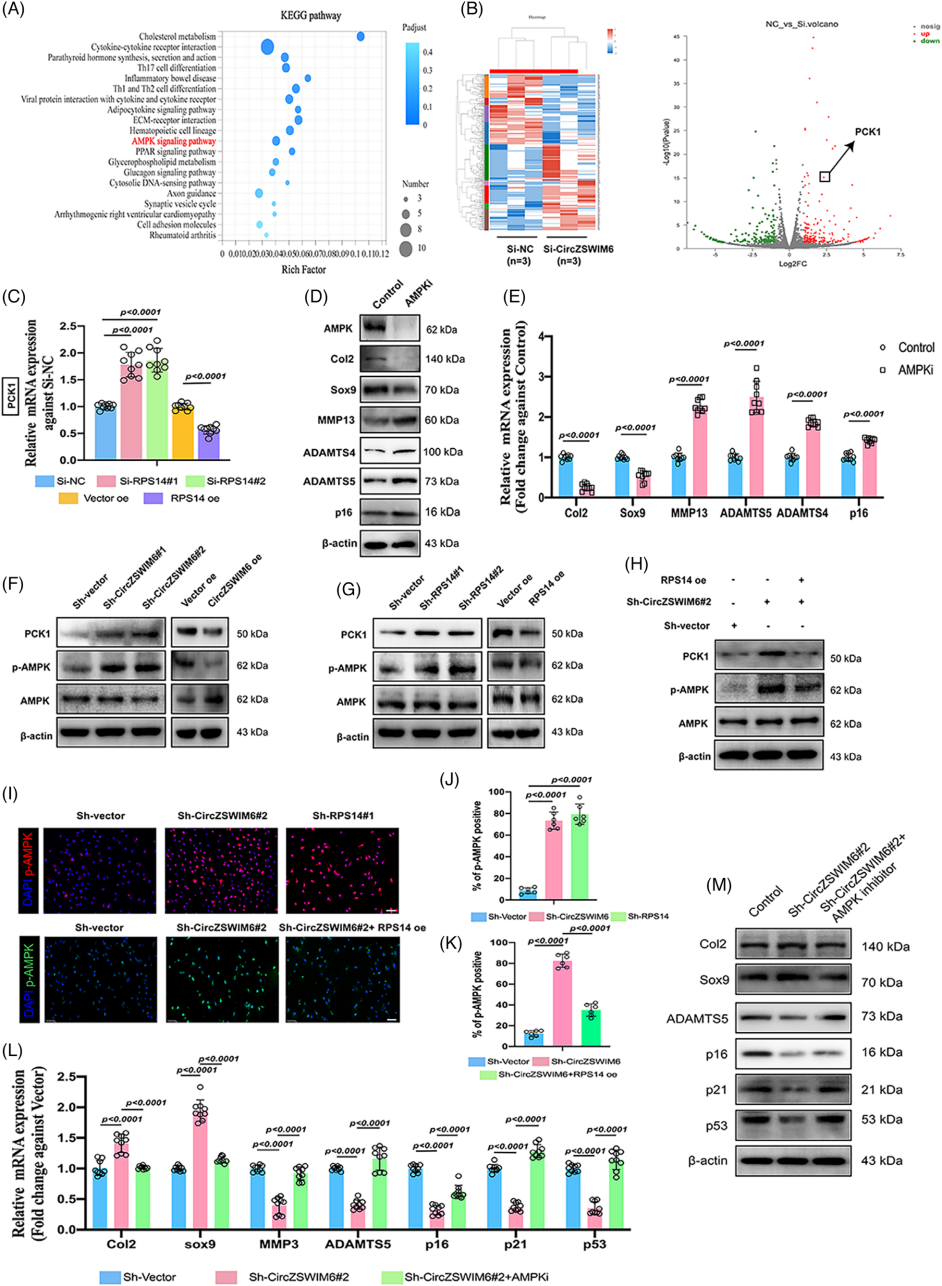
AMP‐activated protein kinase (AMPK) signalling pathway is the downstream of CircZSWIM6‐RPS14 axis: (A) KEGG enrichment after CircZSWIM6 knock‐down; (B) volcano plot of the gene expression differences in CircZSWIM6 knock‐down human chondrocyte; (C) the relative phoenolpyruvate carboxykinase 1 (PCK1) mRNA expression in ribosomal protein S14 (RPS14) knock‐down or overexpression human chondrocyte indicated by quantitative real‐time polymerase chain reaction (RT‐qPCR) (*n* = 3, three donors for three replicates); (D) protein level of AMPK, Col2, Sox9, MMP13, ADAMTS4, ADAMTS5 and p16 after AMPK inhibition; (E) mRNA level of Col2, Sox9, MMP13, ADAMTS4, ADAMTS5 and p16 after AMPK inhibition; (F) the relative PCK1 mRNA expression in RPS14 knock‐down or overexpression human chondrocyte indicated by RT‐qPCR; (G) phosphorylation of AMPK (p‐AMPK), AMPK and PCK1 in human chondrocyte with CircZSWIM6 knock‐down or overexpression and RPS14 knock‐down or overexpression; (H) the rescue effects of RPS14 overexpression on p‐AMPK, AMPK and PCK1 in human chondrocyte indicated by western blot analysis; (I) representative immunofluorescence images of p‐AMPK in human chondrocyte infected with ShCircZSWIM6, ShRPS14 or ShCircZSWIM6 followed by RPS14 overexpression; (J) quantification of p‐AMPK‐positive cells after CircZSWIM6 or RPS14 knock‐down (*n* = 6); (K) quantification of RPS14 overexpression rescue effect on p‐AMPK‐positive staining (*n* = 6); (L) the effect of AMPK inhibition on *Col2*, *Sox9*, *MMP3*, *ADAMTS5*, *p16*, *p21* and, *p53* mRNA expressions after CircZSWIM6 knock‐down indicated by RT‐qPCR (*n* = 3, three donors for three replicates); (M) the effect of AMPK inhibition on Col2, Sox9, ADAMTS5, p16, p21 and p53 protein expressions after CircZSWIM6 knock‐down indicated by western blot; quantitative data shown as mean ± SD; exact *p*‐values are shown in figures. Two‐side unpaired Student's *t*‐test was used for statistical analysis. One‐way ANOVA with Tukey's multiple comparison was used for statistical analysis.

After CircZSWIM6 knock‐down, mRNA‐seq (Majorbio Bio‐pharm Technology Co., Ltd, Shanghai, China) was performed to examine downstream genes. Between the Si‐NC and Si‐CircZSWIM6 HCs (*n* = 3), among a total of 402 different genes, 222 were upregulated and 180 genes were downregulated (Figure 6B). KEGG enrichment analysis showed that CircZSWIM6 inhibition was associated with the AMPK signalling pathway (Figure 6A). Among the downstream genes after CircZSWIM6 knock‐down, we selected *PCK1*, present in a gene cluster related to the AMPK signalling pathway (Figure 6B). Knock‐down or overexpression of *RPS14* enhanced or decreased *PCK1* mRNA expression, respectively, indicating that *PCK1* is a downstream gene of the CircZSWIM6‐RPS14 axis (Figure 6C).

Chondrocytes treated with dorsomorphin (an AMPK inhibitor) exhibited lower AMPK levels (Figure 6D). For ECM metabolism, AMPK inhibition (AMPKi) promoted ECM‐degrading enzymes expression and decreased ECM components (Figure 6D,E). We then investigated whether the CircZSWIM6–RPS14 axis regulates the AMPK signalling pathway. CircZSWIM6 knock‐down increased PCK1 and phosphorylated AMPK (p‐AMPK) expression, whereas CircZSWIM6 overexpression displayed the opposite effect, as indicated by western blot analysis (Figure 6F). RPS14 inhibition increased PCK1 and p‐AMPK expressions, whereas RPS14 overexpression had the opposite effect (Figure 6G). Furthermore, rescue assays showed that RPS14 overexpression after CircZSWIM6 knock‐down reversed the impact on PCK1 and p‐AMPK induced by CircZSWIM6 knock‐down only (Figure 6H). Immunofluorescence images showed that CircZSWIM6 or RPS14 knock‐down promoted p‐AMPK‐positive staining and nuclear translocation (Figure 6I). Compared to the CircZSWIM6 knock‐down group, RPS14 overexpression decreased p‐AMPK‐positive staining and nuclear translocation (Figure 6I). CircZSWIM6 and RPS14 significantly enhanced the nuclear translocation of p‐AMPK, whereas RPS14 overexpression reversed this effect which was induced by CircZSWIM6 inhibition (Figure 6J,K). AMPKi reversed the effects of CircZSWIM6 knock‐down on ECM metabolism and senescence‐associated factors (Figure 6L,M). These data suggested that the CircZSWIM6–RPS14 axis regulates the downstream AMPK signalling pathway. *CircZSWIM6–RPS14 axis regulates the PCK1‐related AMPK signalling pathways to participate in energy homeostasis*.

**FIGURE 7:**
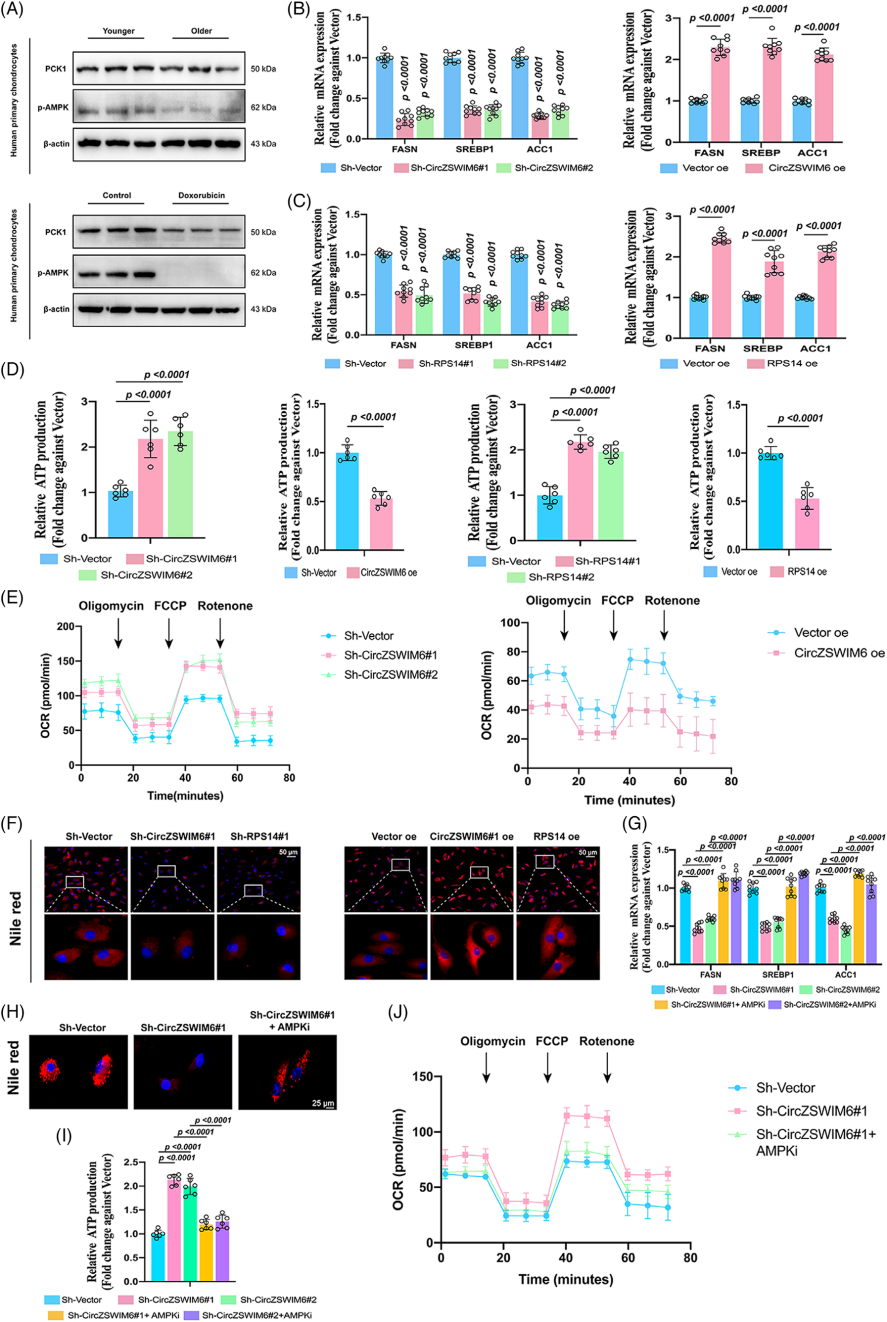
CircZSWIM6–RPS14–PCK1 axis regulates energy metabolism via governing AMP‐activated protein kinase (AMPK) signalling pathway: (A) protein level of p‐AMPK and phoenolpyruvate carboxykinase 1 (PCK1) in the senescence development; (B) mRNA level of fatty acid synthesis–associated genes *FASN*, *SREBP1* and *ACC1* in human chondrocyte with CircZSWIM6 or ribosomal protein S14 (RPS14) knock‐down (*n* = 3, three donors for three replicates); (C) mRNA level of fatty acid synthesis–associated genes *FASN*, *SREBP1* and *ACC1* in human chondrocyte with CircZSWIM6 or RPS14 overexpression (*n* = 3, three donors for three replicates); (D) adenosine triphosphate (ATP) level in human chondrocyte with or without CircZSWIM6 knock‐down or overexpression and RPS14 knockdown or overexpression (*n* = 6); (E) oxygen consumption rates (OCR) in chondrocytes with CircZSWIM6 knock‐down or overexpression; (F) Nile red staining after CircZSWIM6 knock‐down or overexpression and RPS14 knock‐down or overexpression; (G–J) effect of AMPK signalling pathway inhibition after CircZSWIM6 knock‐down on mRNA level of *FASN*, *SREBP1* and *ACC1*, ATP production, OCR rates and Nile red staining; quantitative data shown as mean ± SD; exact *p*‐values are shown in figures. Two‐side unpaired Student's *t*‐test was used for statistical analysis. One‐way ANOVA with Tukey's multiple comparison was used for statistical analysis.

Primary chondrocytes of younger (45–55 years) and older (75–85 years) individuals were obvious, showing that PCK1 and p‐AMPK levels were significantly decreased with the development of the latter group (Figure 7A). Doxo‐induced chondrocytes also showed a similar result using western blot analysis, suggesting that the interconnection of PCK1 and p‐AMPK participated in senescence regulation (Figure 7A).

Energy homeostasis involves ATP production and consumption. Based on the KEGG results, we studied fatty acid synthesis, which requires ATP. Knock‐down of CircZSWIM6 or RPS14 decreased the expression of fatty acid synthesis‐related genes, such as *FASN*, *SREBP1* and *ACC1* (Figure 7B), whereas an overexpression of CircZSWIM6 or RPS14 enhanced the expression of *FASN, SREBP1* and *ACC1* (Figure 7C). CircZSWIM6 or RPS14 knock‐down upregulated ATP production in chondrocytes, whereas CircZSWIM6 or RPS14 overexpression showed an opposite effect (Figure 7D). In addition, OCR analysis also showed increased OCR rate in chondrocytes with CircZSWIM6 knock‐down while decreased after CircZSWIM6 overexpression (Figure 7E). Nile red staining was used to assess lipid synthesis in the chondrocytes. The knock‐down of CircZSWIM6 or RPS14 showed fewer Nile red–positive regions, whereas the overexpression of CircZSWIM6 or RPS14 exhibited more Nile red–positive region than that of the vector‐transfected group (Figure 7F). To verify the effects of AMPKi on energy homeostasis, a rescue experiment was conducted. AMPKi reversed the effects induced by CircZSWIM6 knock‐down, which manifested as fatty acid synthesis associated with *FASN*, *ACC1* and *SREBP1* upregulation, reduction of ATP production, decreased OCR rate and enhancement of the Nile red–positive region (Figure 7F–J). Overall, the CircZSWIM6‐RPS14 axis regulates energy homeostasis through regulating the AMPK signalling transduction.

### 3.9 CircZSWIM6 affects OA pathogenesis in mice

CircZSWIM6 was significantly upregulated in 28‐month‐old mice as indicated by FISH staining of CircZSWIM6 (Figure S4a). Specific CircZSWIM6 or vector AAV (approximately 1 × 10^12^ vg/ml) was injected into the joints of mice to infect cartilage. GFP‐positive AAV integrated into chondrocytes were also observed (Figure S4b). Proteoglycan loss and cartilage damage were clearly observed after CircZSWIM6 infection, as indicated by Safranin‐O‐positive regions, OARSI scores and cartilage thickness quantification (Figure S4c–e). For molecular detection, CircZSWIM6 infection enhanced the expression of senescence markers p16 and p21, SASPs such as CXCL1 and IL‐6, decreasing aggrecan‐positive ECM components (Figure S4f). Quantification of aggrecan, p16, p21, CXCL1 and IL‐6 revealed the same results (Figure S4g–k). Furthermore, we confirmed that CircZSWIM6 overexpression increased RPS14 expression and decreased PCK1 and p‐AMPK levels (Figure S4g–k), as well as the abundance of RPS14, PCK1 and p‐AMPK (Figure S4l–o), which is according to in vitro results.

**FIGURE 8:**
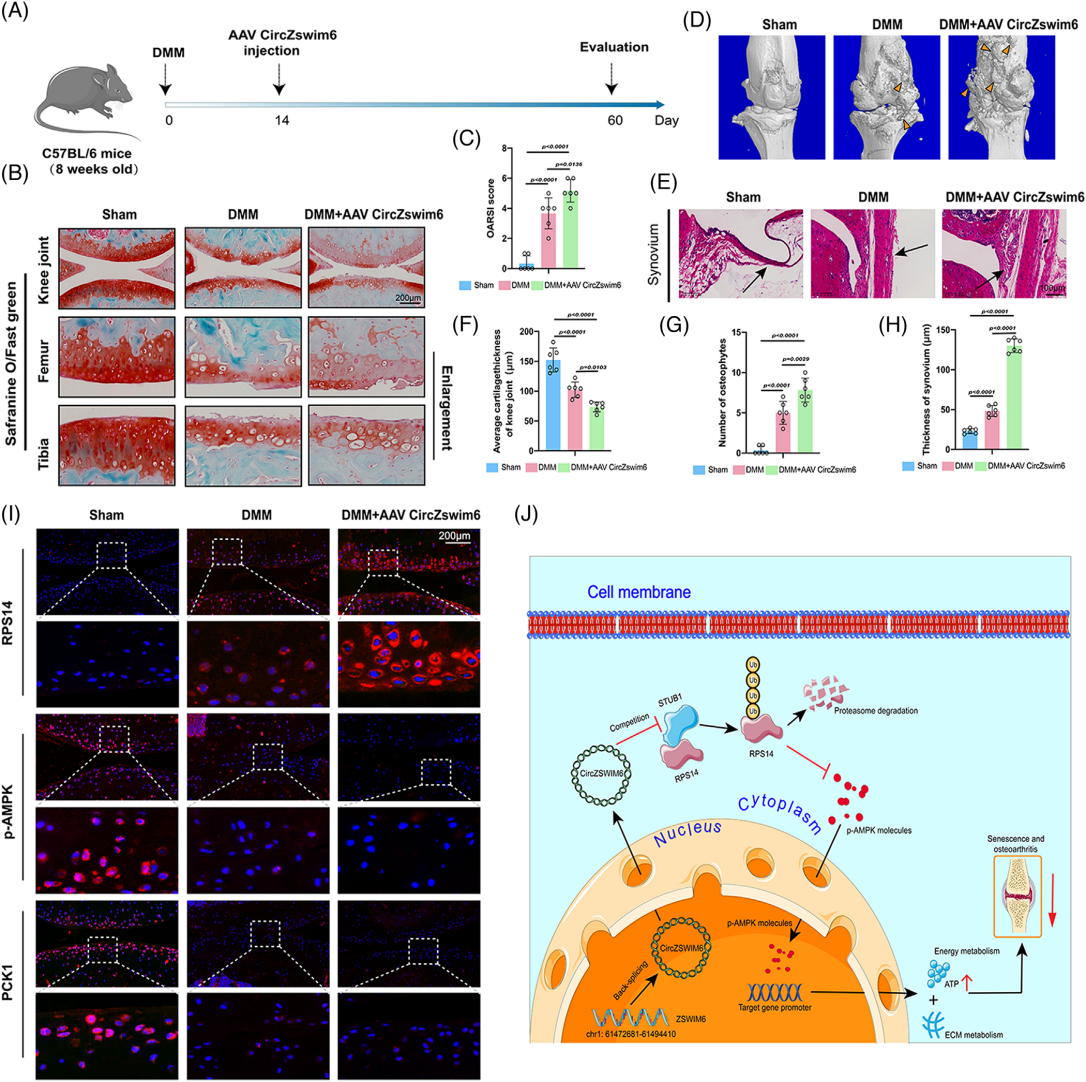
CircZSWIM6 aggravates osteoarthritis (OA) progression in a murine model: (A) a chart for OA model construction, CircZSWIM6 AAV injection and evaluation; (B) representative images of and Safranin‐O/Fast green staining of cartilage in sham, DMM and DMM + AAV CircZSWIM6 groups; (C) Osteoarthritis Research Society International (OARSI) grade system used for cartilage degradation evaluation in the three groups (*n* = 6); (D) micro‐CT 3D images and osteophytes in knee joints (*n* = 6); (E) H&E staining of synovium (*n* = 6); (F–H) quantification of synovium thickness, osteophytes number and cartilage thickness (*n* = 6); (I) representative immunofluorescence images of ribosomal protein S14 (RPS14), p‐AMPK and phoenolpyruvate carboxykinase 1 (PCK1) of articular cartilage in the three groups; (J) Graphical abstract of mechanism of CircZSWIM6 in OA and cellular senescence development; quantitative data shown as mean ± SD; exact *p*‐values are shown in figures. Two‐side unpaired Student's *t*‐test was used for statistical analysis. One‐way ANOVA with Tukey's multiple comparison was used for statistical analysis.

For the post‐traumatic model, CircZSWIM6 AAV was injected after the DMM operation (Figure 8A). Animals that underwent DMM operation and CircZSWIM6 overexpression showed a decrease according to Safranin‐O‐positive staining (Figure 8B). Interestingly, CircZSWIM6 AAV injection after DMM resulted in the most dramatic loss of proteoglycan among the three groups (Figure 8B). The OARSI scoring system also indicated that CircZSWIM6 accelerated cartilage disruption (Figure 8C). Quantification of articular surface thickness yielded similar results (Figure 8D). The number of osteophytes indirectly reflected the degree of OA, and micro‐CT was used to observe osteophytes after CircZSWIM6 infection. More osteophytes were observed in the DMM + CircZSWIM6 group than in the sham and DMM groups (Figure 8D). The quantification of the number of osteophytes was consistent with the above results (Figure 8G). In addition, DMM and CircZSWIM6 overexpression promoted synovial hyperplasia (Figure 8E). Quantification of synovial thickness showed that CircZSWIM6 overexpression dramatically enhanced synovial hyperplasia, resulting in OA progression (Figure 8H). Representative immunofluorescence staining showed that DMM operation and CircZSWIM6 overexpression upregulated RPS14 expression and downregulated PCK1 and p‐AMPK expressions, whereas animals that received DMM operation and CircZSWIM6 AAV injection showed more severe results than animals that received DMM operation only (Figure 8I). At the molecular level, GFP‐positive CircZSWIM6 was successfully integrated into chondrocytes (Figure S5a). ECM components, such as Col2, were significantly decreased after DMM and CircZSWIM6 overexpression. However, senescence markers, such as p16 and p21, and SASP molecules, such as IL‐6 and CXCL1, were increased when CircZSWIM6 was overexpressed after DMM operation (Figure S5b–g). These data demonstrated that CircZSWIM6 and RPS14 participate in OA and senescence development in mice. The underlying mechanisms are illustrated in Figure 8J.

## 4 DISCUSSION

The predominant phenomenon in OA is an imbalance in cartilage matrix metabolism, including the upregulation of catabolism and downregulation of anabolism, which leads to chondrocyte matrix degradation.^30^ Chondrocyte senescence is usually considered a key regulator of OA development.^31^ Moreover, energy metabolism supports chondrocyte growth, whereas energy disorders, including lower ATP production, may accelerate OA progression. However, the mechanisms underlying matrix and energy metabolism disorders caused by chondrocyte senescence have not yet been thoroughly studied. Herein, we clarified that the senescence‐mediated upregulation of CircZSWIM6 leads to an imbalance in matrix and energy metabolism in chondrocytes. An exciting new potential target for treating OA was identified by our study.

Numerous studies have focused on gene therapy for the treatment of senescence‐associated diseases. CircRNAs are widely used for this purpose because of their various biological functions.^32^ The currently reported mechanisms of circRNAs can be classified into the following three types. First, circRNAs regulate gene expression and coding. Second, circRNAs sponge miRNAs to regulate downstream genes. Finally, circRNAs bind to proteins to form circRNA‐protein complexes that are involved in disease development. We previously confirmed that CircSERPINE2 regulates the ERG gene by competing endogenous RNA, alleviating OA development.^24^ Recently, we found that CircRNA could bind specific protein in OA progression.^5^ The mechanisms by which RNA‐binding proteins interact with circRNAs have received extensive attention. Chondrocyte senescence is a key regulator in OA. This study aims to describe how CircZSWIM6 stabilizes RPS14 to disrupt the matrix and energy metabolism. We found the upregulation of CircZSWIM6 expression in ageing chondrocytes. ECM regulation is important for the development of OA. Senescent chondrocytes exhibit reduced ability to construct the ECM, thereby accelerating OA progression. However, our findings indicated that CircZSWIM6 knock‐down partially reversed the damage caused by chondrocyte senescence. These data show that CircZSWIM6 upregulation in chondrocytes governs the regulation of ECM metabolism to promote age‐related OA development. To confirm the function of CircZSWIM6 in age‐related OA development, we explored the underlying mechanisms by which CircZSWIM6 contributes to the progression of aging. Recently, Frydman et al. reported that ribosome pausing is a key event in senescence development,^33^ suggesting that protein function in ribosomes may play important roles in this process. RPS14 has been reported to promote malignant tumour metastasis and is associated with blood diseases.^34^ However, the role of RPS14 in OA‐associated senescence remains unclear. In this study, we found that RPS14 acts as an RNA‐binding protein for CircZSWIM6 in vitro. An interaction between RPS14 and CircZSWIM6 was also observed. Post‐translational modifications regulate protein activity in many diseases.^35^ CircZSWIM6 affected RPS14 protein levels but did not affect *RPS14* mRNA expression, which suggesting that CircZSWIM6 may affect post‐translational modifications of RPS14. Ubiquitylation is a major post‐translational modification that plays an important role in physiological processes including cellular function, cellular responses, apoptosis, and signal transduction.^36^ The ubiquitin–proteasome pathway is a classical degradation pathway. Our results showed that the proteasome pathway inhibitor Borz affected RPS14 protein levels. CircZSWIM6 knockdown increased RPS14 ubiquitination, whereas CircZSWIM6 overexpression decreased ubiquitination, suggesting that CircZSWIM6 stabilized RPS14 by inhibiting the ubiquitin–proteasome pathway. Exploring how CircZSWIM6 affects RPS14 through the ubiquitin–proteasome system, we found that the E3 ligase STUB1 interacts with RPS14 to promote its degradation. We demonstrated that CircZSWIM6 inhibition enhanced the interaction between STUB1 and RPS14, whereas CircZSWIM6 overexpression showed the opposite effect. Moreover, STUB1 overexpression accelerated RPS14 ubiquitination. These data suggested that CircZSWIM6 occupies the binding site of STUB1 on RPS14. Employing an acetyl‐deficient mutant, K125 was verified to be a major ubiquitylation site of RPS14 when STUB1 was overexpressed. Furthermore, K11/K48 polyubiquitylation types participate in proteasomal degradation.^37^, ^38^ Hence, in this study, we performed different ubiquitin chain plasmids transfections and found that the K11 type was associated with STUB1‐induced polyubiquitylation of RPS14. In summary, CircZSWIM6 stabilizes RPS14 by competitively binding to the ubiquitination site of STUB1 on RPS14, thereby reducing the degradation of the ubiquitin–proteasome pathway.

Chondrocyte senescence is characterized by an imbalance involving matrix and energy metabolism. The energy supply is very important for cell growth; however, less ATP is produced during chondrocyte senescence.^15^ The AMPK signalling pathway is associated with ATP balance and is significantly downregulated in senescent cells.^39^ Through mRNA‐seq, we found that CircZSWIM6 knock‐down upregulated AMPK signal transduction. Furthermore, we confirmed that the knock‐down or overexpression of RPS14 increased and decreased p‐AMPK expression, respectively. Thus, we hypothesized that CircZSWIM6–RPS14 regulates AMPK signalling pathways in ageing chondrocytes to regulate energy metabolism. Among the downstream genes related to the AMPK signalling pathway, we found that PCK1 regulates this pathway, which is consistent with previous evidence.^40^ The role of CircZSWIM6 in energy metabolism was also investigated. CircZSWIM6 and RPS14 knock‐downs promoted ATP production in HCs and reduced fatty acid synthesis, which consumes ATP. CircZSWIM6 knock‐down increased the OCR of chondrocytes. We also found that p‐AMPK levels were significantly decreased in ageing chondrocytes. In accordance with RNA‐seq, these results demonstrated that CircZSWIM6‐RPS14 regulates the transduction of AMPK signals for energy metabolism.

Overall, we confirmed the role of a circRNA, CircZSWIM6, that is upregulated in older chondrocytes. CircZSWIM6 stabilized RPS14 to regulate ECM metabolism and promote cartilage matrix degradation. Mechanistically, CircZSWIM6 competitively bound to the STUB1 site on RPS14 to prevent proteasomal degradation. Additionally, CircZSWIM6 stabilized RPS14 to inhibit the activation of AMPK signalling, thereby disturbing the energy metabolism balance. We believe that our findings provide a potential target for the future clinical treatment of senescence‐associated diseases, including OA.

## CONFLICT OF INTEREST

The authors declare no conflict of interest.

## Supporting information

Supporting Information
