# Supplementary material for: CircZSWIM6 mediates dysregulation of ECM and energy homeostasis in ageing chondrocytes through RPS14 post‐translational modification
Source: Clin Transl Med. 2023 Jan 5;13(1):e1158. doi: 10.1002/ctm2.1158 (PMC9816529; doi:10.1002/ctm2.1158)
Supplement: Supplementary file 1 — Supporting Information [file CTM2-13-e1158-s001.docx]

**Supplementary materials**

**CircZSWIM6 mediates dysregulation of ECM and energy homeostasis in aging chondrocytes through RPS14 post-translational modification**

**Authors and Affiliations:**

Zhe Gong^#,1^, Kefan Wang^#,1^, Junxing Chen^#,1^, Jinjin Zhu^1^, Zhenghua Feng^1^, Chenxin Song^1^, Zheyuan Zhang^1^, Haoming Wang^1^ , Shunwu Fan^*,1^, Shuying Shen^*,1^, & Xiangqian Fang^*,1^

1.Department of Orthopaedic Surgery, Sir Run Run Shaw Hospital, Medical College of Zhejiang University & Key Laboratory of Musculoskeletal System Degeneration and Regeneration Translational Research of Zhejiang Province

Sir Run Run Shaw Institute of Clinical Medicine of Zhejiang University 3 East Qingchun Road, Hangzhou, Zhejiang Province, China, 310016

**Supplementary Figures**

**Supplementary Fig. S1**


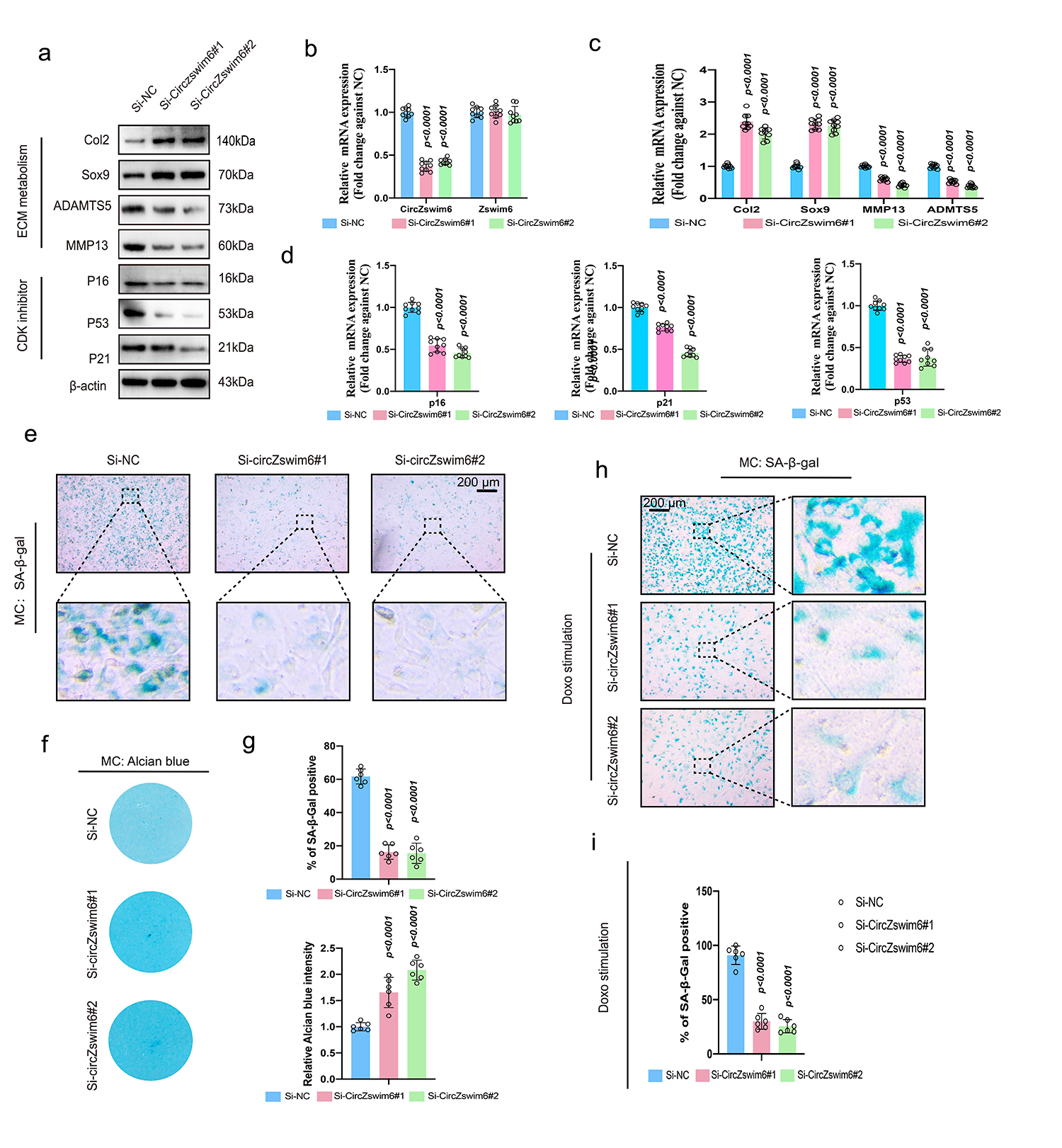


**Supplementary Fig. S1 CircZSWIM6 regulates ECM metabolism and senescent phenotypes in mouse chondrocyte.** (a) Western blot analysis of Col2, Sox9, ADAMTS5, MMP13, p16, p21 and p53 expression level in mouse chondrocyte after CircZSWIM6 knockdown. (b) Mouse chondrocyte transfected with two CircZSWIM6 siRNA. Knockdown efficiency indicated by RT-qPCR. No change of *Zswim6* host gene expression was detected. (c, d) mRNA level of *Col2, Sox9, MMP13, MMP3, ADAMTS5, p16, p21, p53* in mouse chondrocyte with CircZSWIM6 knockdown (n=3, 3 donors for three replicates). (e) Representative images of SA-β-Gal in mouse chondrocyte with CircZSWIM6 knockdown. (f) Alcian blue staining after CircZSWIM6 knockdown. (g) Quantification of SA-β-Gal positive and Alcian blue staining after CircZSWIM6 knockdown (n=6). (h) SA-β-Gal staining of mouse chondrocyte after Doxo stimulation with or without CircZSWIM6 knockdown. (i) Quantification of SA-β-Gal positive staining after Doxo stimulation with or without CircZSWIM6 knockdown (n=6). Quantitative data shown as mean ± s.d. Exact p-values are shown in figures. Two-side unpaired Student’s *t*-test was used for statistical analysis. One-way ANOVA with Turkey’s multiple comparison was used for statistical analysis.

**Supplementary Fig S2**


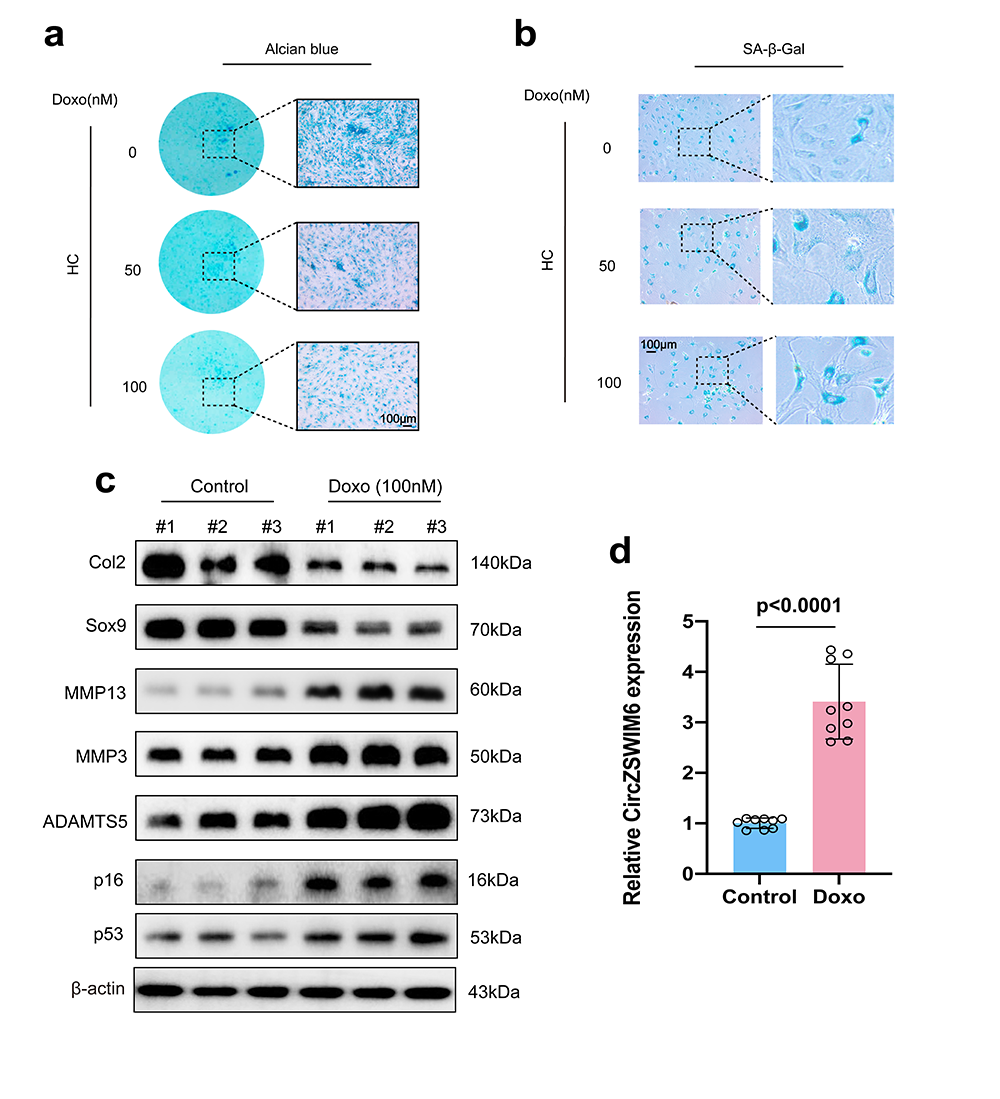


**Supplementary Fig. S2 Doxo stimulation induces chondrocyte senescence phenotypes and promoted CircZSWIM6 upregulation.** (a, b) Representative images of Alcian blue and SA-β-Gal staining after different concentration of Doxo simulation. (c) Col2, Sox9, MMP13, MMP3, ADAMTS5, p16, and p53 proteins expression in chondrocytes with or without Doxo (100 nM) simulation. (d) CircZSWIM6 expression in chondrocytes treated with Doxo (100 nM) indicated by RT-qPCR (n=3, 3 donors for three replicates). Quantitative data shown as mean ± s.d. Exact p-values are shown in figures. Two-side unpaired Student’s *t*-test was used for statistical analysis. One-way ANOVA with Turkey’s multiple comparison was used for statistical analysis.

**Supplementary Fig S3**


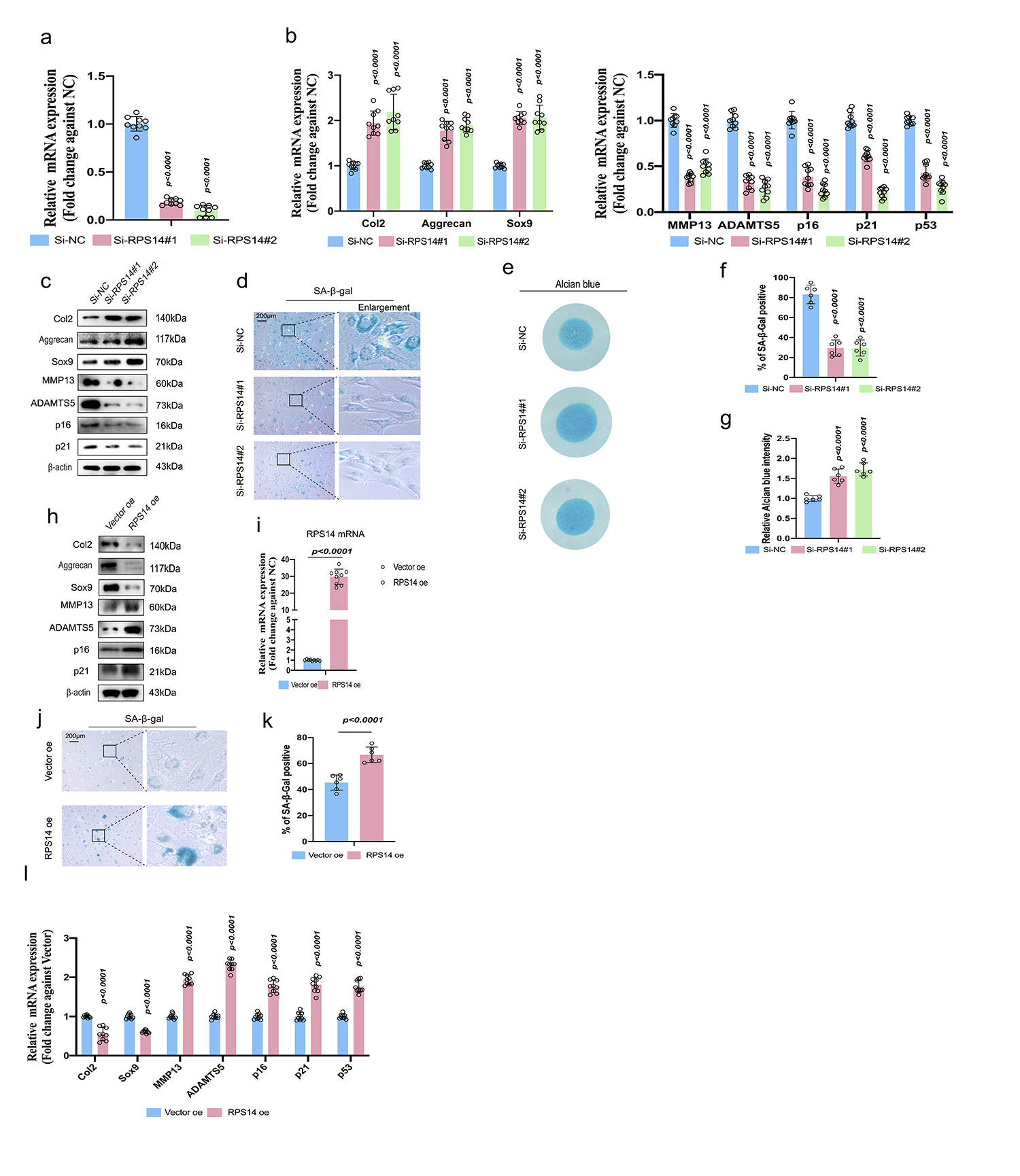


**Supplementary Fig. S3 RPS14 regulates ECM metabolism and senescent phenotypes.** (a) RPS14 knockdown efficiency indicated by RT-qPCR (n=3, 3 donors for three replicates). (b) mRNA level of *Col2, Aggrecan, Sox9, MMP13, ADAMTS5, p16, p21* and *p53* after RPS14 knockdown (n=3, 3 donors for three replicates). (c) Western blot result of Col2, Aggrecan, Sox9, MMP13, ADAMTS5, p16 and p21 expression level after RPS14 knockdown. (d) Representative images of SA-β-Gal staining after RPS14 knockdown. (e) Alcian blue staining after RPS14 knockdown. (f, g) Quantification of SA-β-Gal positive and Alcian blue staining after RPS14 knockdown (n=6). (h) Col2, Aggrecan, Sox9, MMP13, ADAMTS5, p16, p21 protein expression level after RPS14 overexpression. (i) RPS14 overexpression efficiency indicated by RT-qPCR (n=3, 3 donors for three replicates). (j) Representative images of SA-β-Gal staining after RPS14 overexpression. (k) Quantification of SA-β-Gal positive staining after RPS14 overexpression (n=6). (l) Relative mRNA expression of *Col2, Sox9, MMP13, ADAMTS5, p16* and *p21* after RPS14 overexpression (n=3, 3 donors for three replicates). Quantitative data shown as mean ± s.d. Exact p-values are shown in figures. Two-side unpaired Student’s *t*-test was used for statistical analysis. One-way ANOVA with Turkey’s multiple comparison was used for statistical analysis.

**Supplementary Fig S4**


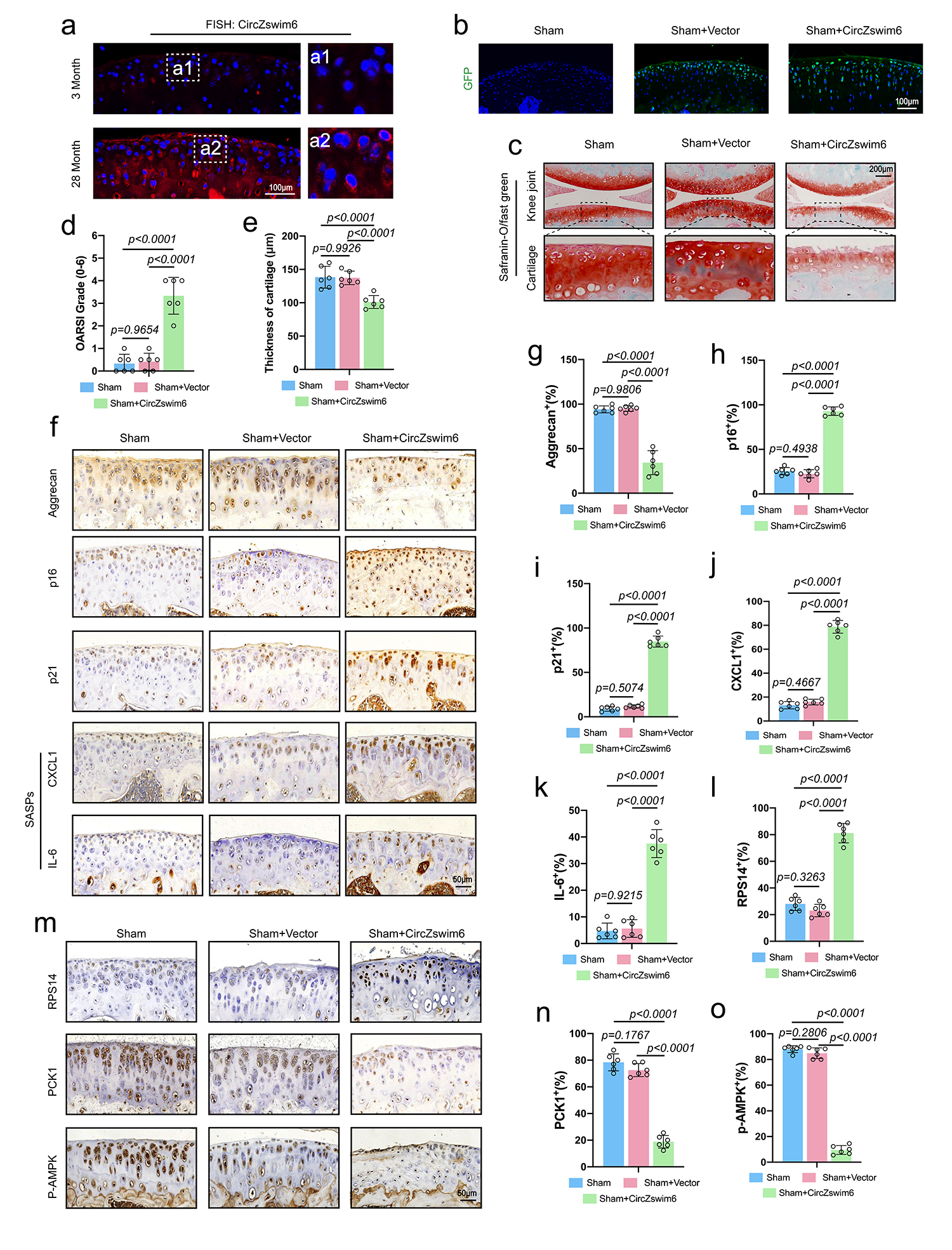


**Supplementary Fig. S4 CircZswim6 caused senescence and OA phenotypes in a non-traumatic model.** (a) FISH staining of CircZswim6 in chondrocyte of 3 month-old and 28 month-old mice. (b) GFP positive AAV integrates with chondrocytes in mice joints. (c) Representative images of Safranin O/Fast green staining of cartilage in Sham, Sham+Vector, and Sham+CircZswim6 groups. (d) OARSI grade system for cartilage degradation evaluation in the three groups (n=6). (e) Quantification of thickness of cartilage in the three groups (n=6). (f) Representative images of Aggrecan, p16, p21, CXCL1, and IL-6 in the three groups. (g-k) Quantification of Aggrecan, p16, p21, CXCL1, and IL-6 in the three groups (n=6). (m) Representative images of RPS14, PCK1, and p-AMPK in the three groups. (l, n, o) Quantification of RPS14, PCK1, and p-AMPK in the three groups (n=6). Quantitative data shown as mean ± s.d. Exact p-values are shown in figures. Two-side unpaired Student’s *t*-test was used for statistical analysis. One-way ANOVA with Turkey’s multiple comparison was used for statistical analysis.

**Supplementary Fig S5**


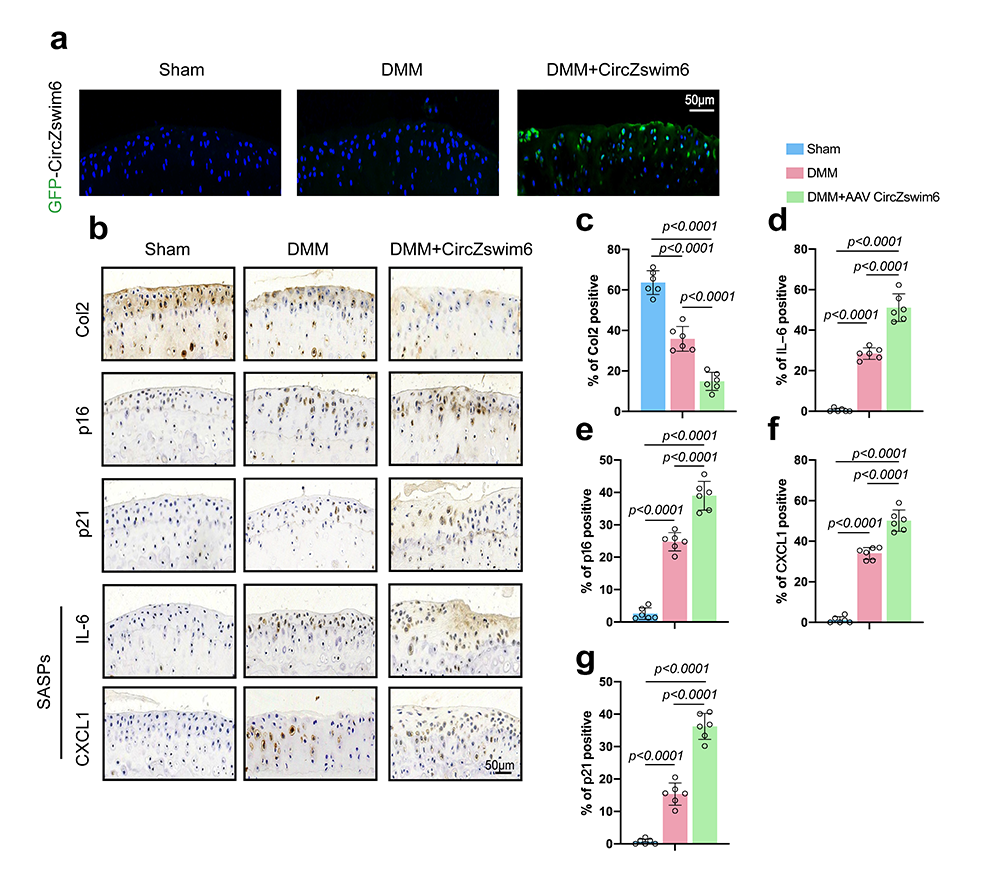


**Supplementary Fig. S5 CircZSwim6 accelerates senescent phenotypes in a DMM models.** (a) Representative immunofluorescence images shows that GFP-CircZswim6 integrates with chondrocytes. (b) Molecular detection of Col2, p16, p21, IL-6, and CXCL1 in chondrocyte in DMM induced murine models. (c-g) Quantifications of Col2, p16, p21, IL-6, and CXCL1 positive cells.

**Supplementary Tables**

**Table S1**

| Primers for qPCR | |
| --- | --- |
| ZSWIM6 mRNA | F:AAGCGGCTGCGTAGACAAC  R:GGCTCCGATTGTATTGCAGGT |
| CircZSWIM6 (hsa) | F:TCGGAGCCAGAAACTGTTTG  R:TGCGGATGCGGTATAAAGACA |
| Zswim6(mu) mRNA | F:CCCTCTGGCATGAACACGTC  R:TCTGCTGTTGTCGTCGTAATG |
| CircZswim6 (mmu) | F:CCCTCTGGCATGAACACGTC  R:TCTGCTGTTGTCGTCGTAATG |
| Human MMP13 | F:TCGGCCACTCCTTAGGTCTT  R:AAGTGGCTTTTGCCGGTGTA |
| Human MMP3 | F:CCTACAAGGAGGCAGGCAAG  R:CCCGTCACCTCCAATCCAAG |
| Human ADAMTS5 | F:GGGCACTGGCTACTATGTGG  R:CGTCACAGCCAGTTCTCACA |
| Human ADAMTS4 | F:GTCCCATGTGCAACGTCAAG  R:ATGCGGCCATCTTGTCATCT |
| Human Sox9 | F: GCTCTGGAGACTTCTGAACGA  R: CCGTTCTTCACCGACTTCCT |
| Human col2 | F: ATGACAATCTGGCTCCCAAC  R: GAACCTGCTATTGCCCTC |
| Human RPS14 | F: CCATGTCACTGATCTTTCTGGC  R: TCATCTCGGTCTGCCTTTACC |
| Human Aggrecan | F:GTGCCTATCAGGACAAGGTCT  R: GATGCCTTTCACCACGACTTC |
| Human p16 | F: GATCCAGGTGGGTAGAAGGTC  R: CCCCTGCAAACTTCGTCCT |
| Human p21 | F: TGTCCGTCAGAACCCATGC  R: AAAGTCGAAGTTCCATCGCTC |
| Human p53 | F: CAGCACATGACGGAGGTTGT  R: TCATCCAAATACTCCACACGC |
| Human PCK1 | F: AAAACGGCCTGAACCTCTCG  R: ACACAGCTCAGCGTTATTCTC |
| Human ACC1 | F: ATGTCTGGCTTGCACCTAGTA  R: CCCCAAAGCGAGTAACAAATTCT |
| Human FASN | F: CCGAGACACTCGTGGGCTA  R: CTTCAGCAGGACATTGATGCC |
| Human SREBP1 | F: ACAGTGACTTCCCTGGCCTAT  R: GCATGGACGGGTACATCTTCAA |
| Human actin | F:AGAGCTACGAGCTGCCTGAC  R:AGCACTGTGTTGGCGTACAG |
| Mouse MMP13 | F:CAAGCAGTTCCAAAGGCTACA  R:TAGGGCTGGGTCACACTTCT |
| Mouse ADAMTS5 | F:ATGCAGCCATCCTGTTCACC  R:AAGGCCAAGTAGATGCCCAATTT |
| Mouse Sox9 | F:TAATTCCCCAGGCTCTTGGAT  R:GCAGCCGGGATTTAAGGCTC |
| Mouse Col2 | F:CACGCATGAGCCGAAGCTA  R:GGGTTTCCACGTCTCACCA |
| Mouse p16 | F:CGCAGGTTCTTGGTCACTGT  R:TGTTCACGAAAGCCAGAGCG |
| Mouse p21 | F:CCTGGTGATGTCCGACCTG  R:CCATGAGCGCATCGCAATC |
| Mouse p53 | F:CTCTCCCCCGCAAAAGAAAAA  R:CGGAACATCTCGAAGCGTTTA |
| Mouse actin | F:AGCCATGTACGTAGCCATCC  R:CTCTCAGCTGTGGTGGTGAA |
| SiRNA sequence | |
| Human CircZSWIM6 si-1 | ATGAGCTGGGTTTCCACTT |
| Human CircZSWIM6 si-2 | GGGATGAGCTGGGTTTCCA |
| Human CircZSWIM6 si-3 | TCTGGGATGAGCTGGGTTT |
| Mouse CircZSWIM6 Si-1 | AGGTAGCTAGCTCCCGTCT |
| Mouse CircZSWIM6 Si-2 | GTAGCTAGCTCCCGTCTTC |
| Mouse CircZSWIM6 Si-3 | AGCTAGCTCCCGTCTTCGT |
| Human RPS14 si-1 | GCCATATCTTTGCATCCTT |
| Human RPS14 si-2 | CCATGTCACTGATCTTTCT |
| ShRNA | |
| CircZSWIM6 shRNA-1 | F:CCGGATGAGCTGGGTTTCCACTTCTCGAGAAGTGGAAACCCAGCTCATTTTTTC  R:TACTCGACCCAAAGGTGAAGAGCTCTTCACCTTTGGGTCGAGTAAAAAAGTTAA |
| CircZSWIM6 shRNA-2 | F:CCGGGGGATGAGCTGGGTTTCCACTCGAGTGGAAACCCAGCTCATCCCTTTTTC  R:CCCTACTCGACCCAAAGGTGAGCTCACCTTTGGGTCGAGTAGGGAAAAAGTTAA |
| CircZSWIM6 shRNA-3 | F:CCGGTCTGGGATGAGCTGGGTTTCTCGAGAAACCCAGCTCATCCCAGATTTTTC  R:AGACCCTACTCGACCCAAAGAGCTCTTTGGGTCGAGTAGGGTCTAAAAAGTTAA |
| RPS14 shRNA-1 | F:CCGGGCCATATCTTTGCATCCTTCTCGAGAAGGATGCAAAGATATGGCTTTTTC  R:CGGTATAGAAACGTAGGAAGAGCTCTTCCTACGTTTCTATACCGTTAA |
| RPS14 shRNA-2 | F:CCGGCCATGTCACTGATCTTTCTCTCGAGAGAAAGATCAGTGACATGGTTTTTC  R: GGTACAGTGACTAGAAAGAGAGCTCTCTTTCTAGTCACTGTACCTTAA |
| Probes for FISH | |
| Cy3-Has circZSWIM6 | 5‘-Cy3-CTCAAGTGGAAACCCAGCTCATCCCAG -3’ |
| RNA pulldown probe | |
| CircZSWIM6 pull-down probe | AAGTGGAAACCCAGCTCATC |

**Table S2. Descriptive characteristics of human cartilage samples with different age. BMI: Body mass index.**

| NM | Gender | Age (year) | Height (m) | Weight (kg) | BMI (kg/m2) |
| --- | --- | --- | --- | --- | --- |
| 1 | Male | 53 | 1.70 | 76.6 | 26.5 |
| 2 | Female | 55 | 1.55 | 51.9 | 21.6 |
| 3 | Male | 45 | 1.72 | 70.0 | 23.7 |
| 4 | Male | 47 | 1.64 | 69.5 | 25.8 |
| 5 | Female | 48 | 1.63 | 65.0 | 24.5 |
| 6 | Female | 51 | 1.53 | 54.0 | 23.1 |
| 7 | Female | 50 | 1.55 | 65.0 | 27.1 |
| 8 | Female | 47 | 1.56 | 55.0 | 22.6 |
| 9 | Male | 51 | 1.71 | 85.0 | 29.1 |
| 10 | Male | 52 | 1.63 | 68.0 | 25.6 |
| 11 | Female | 75 | 1.52 | 56.0 | 24.2 |
| 12 | Female | 78 | 1.55 | 65.0 | 27.1 |
| 13 | Female | 81 | 1.58 | 65.0 | 26 |
| 14 | Male | 83 | 1.70 | 68.4 | 23.7 |
| 15 | Female | 85 | 1.60 | 80.0 | 31.3 |
| 16 | Female | 75 | 1.55 | 62.3 | 25.9 |
| 17 | Male | 77 | 1.72 | 80.0 | 27 |
| 18 | Female | 80 | 1.62 | 71.0 | 27.1 |
| 19 | Female | 78 | 1.55 | 51.7 | 21.5 |
| 20 | Male | 78 | 1.65 | 69.0 | 25.3 |

**Table S3. Top 20 CircZSWIM6-binding proteins identified by mass spectrometry (ranked by prot_score).**

| Number | Accession | Description | prot_score |
| --- | --- | --- | --- |
| 1 | P07437 | Myosin-9 OS=Homo sapiens OX=9606 GN=MYH9 PE=1 SV=4 | 608 |
| 2 | P07437 | Tubulin beta chain OS=Homo sapiens OX=9606 GN=TUBB PE=1 SV=2 | 252 |
| 3 | P08238 | Heat shock protein HSP 90-beta OS=Homo sapiens OX=9606 GN=HSP90AB1 PE=1 SV=4 | 175 |
| 4 | P67936 | Tropomyosin alpha-4 chain OS=Homo sapiens OX=9606 GN=TPM4 PE=1 SV=3 | 165 |
| 5 | P60709 | Actin, cytoplasmic 1 OS=Homo sapiens OX=9606 GN=ACTB PE=1 SV=1 | 161 |
| 6 | Q16643 | Drebrin OS=Homo sapiens OX=9606 GN=DBN1 PE=1 SV=4 | 160 |
| 7 | P25705 | ATP synthase subunit alpha, mitochondrial OS=Homo sapiens OX=9606 GN=ATP5F1A PE=1 SV=1 | 156 |
| 8 | P22087 | rRNA 2~-O-methyltransferase fibrillarin OS=Homo sapiens OX=9606 GN=FBL PE=1 SV=2 | 153 |
| 9 | P78527 | DNA-dependent protein kinase catalytic subunit OS=Homo sapiens OX=9606 GN=PRKDC PE=1 SV=3 | 132 |
| 10 | P62269 | 40S ribosomal protein S18 OS=Homo sapiens OX=9606 GN=RPS18 PE=1 SV=3 | 130 |
| 11 | P11142 | Heat shock cognate 71 kDa protein OS=Homo sapiens OX=9606 GN=HSPA8 PE=1 SV=1 | 128 |
| 12 | Q9UPN3 | Microtubule-actin cross-linking factor 1, isoforms 1/2/3/5 OS=Homo sapiens OX=9606 GN=MACF1 PE=1 SV=4SV=3 | 125 |
| 13 | Q9Y4W2 | Ribosomal biogenesis protein LAS1L OS=Homo sapiens OX=9606 GN=LAS1L PE=1 SV=2 | 120 |
| 14 | Q14204 | Cytoplasmic dynein 1 heavy chain 1 OS=Homo sapiens OX=9606 GN=DYNC1H1 PE=1 SV=5 | 120 |
| 15 | Q7Z406 | Myosin-14 OS=Homo sapiens OX=9606 GN=MYH14 PE=1 SV=2 | 117 |
| 16 | P49411 | Elongation factor Tu, mitochondrial OS=Homo sapiens OX=9606 GN=TUFM PE=1 SV=2 | 117 |
| 17 | Q8NE71 | ATP-binding cassette sub-family F member 1 OS=Homo sapiens OX=9606 GN=ABCF1 PE=1 SV=2 | 115 |
| 18 | P62263 | 40S ribosomal protein S14 OS=Homo sapiens OX=9606 GN=RPS14 PE=1 SV=3 | 110 |
| 19 | P04350 | Tubulin beta-4A chain OS=Homo sapiens OX=9606 GN=TUBB4A PE=1 SV=2 | 109 |
| 20 | Q6RW13 | Type-1 angiotensin II receptor-associated protein OS=Homo sapiens OX=9606 GN=AGTRAP PE=1 SV=1 | 109 |

**Table S4**

A diagram of animals used in a non-traumatic model

A diagram of animals used in a DMM model

**Table S5. Antibodies information**

| Antibodies | Company | Catalog # | Application/Dilution |
| --- | --- | --- | --- |
| Col2 | Bioss | bs-5881R | WB (1:1000) |
| Aggrecan | Abcam | ab3778 | WB (1:1000) |
| ADAMTS5 | Abcam | ab41037 | WB (1:250) |
| MMP3 | Abcam | ab52915 | WB (1:2000) |
| MMP13 | Abcam | ab39012 | WB (1:5000) IF (1:100) |
| P16 | Santa Cruz | Sc-51243 | IF (1:100); IHC (1:50) |
| P21 | Santa Cruz | Sc-6246 | WB (1:200); IHC (1:50); IF (1:50) |
| P53 | Proteintech | 60283-2-lg | WB (1:5000) |
| P16 | Proteintech | 10883-1-AP | WB (1:1000) |
| Beta actin | HUABIO | M1210-2 | WB (1:10000) |
| Sox9 | Abcam | Ab182579 | WB (1:1000) |
| Aggrecan | Proteintech | 13880-1-AP | IHC (1:100); IF (1:250) |
| RPS14 | Santa Cruz | sc293478 | WB (1:1000); IF (1:250); IP(1:50); IHC (1:50) |
| CXCL1 | Proteintech | 12335-1-AP | IHC (1:100) |
| IL-6 | Abcam | Ab9324 | IHC (1:100) |
| STUB1 | Abcam | Ab109103 | WB (1:1000); IF (1:100) |
| AMPK | Abcam | Ab32047 | WB (1:1000) |
| p-AMPK | Affinity | AF3423 | WB (1:1000); IHC (1:100); IF (1:100) |
| Ubiquitin | Abcam | Ab134953 | WB (1:1000) |
| PCK1 | Abcam | Ab133603 | WB (1:1000) |
| Myc-tag | Abcam | Sc-40 | WB (1:200); IP (1:100) |
| Flag-tag | MBL | M185-3L | WB (1:10000) IP (1:100) |
| HA-tag | Santa Cruz | Sc-7392 | WB (1:200) IP (1:100) |
| PCK1 | Proteintech | 16754-1-AP | IF (1:250) |
